# Interaction of *Phytophthora sojae* Effector Avr1b With E3 Ubiquitin Ligase GmPUB1 Is Required for Recognition by Soybeans Carrying *Phytophthora* Resistance *Rps1*-b and *Rps1*-k Genes

**DOI:** 10.3389/fpls.2021.725571

**Published:** 2021-10-06

**Authors:** Shan Li, Regina Hanlon, Hua Wise, Narinder Pal, Hargeet Brar, Chunyu Liao, Hongyu Gao, Eli Perez, Lecong Zhou, Brett M. Tyler, Madan K. Bhattacharyya

**Affiliations:** ^1^Department of Agronomy, Iowa State University, Ames, IA, United States; ^2^School of Plant and Environmental Sciences, Virginia Tech, Blacksburg, VA, United States; ^3^Center for Quantitative Life Sciences and Department of Botany and Plant Pathology, Oregon State University, Corvallis, OR, United States

**Keywords:** soybean U-box protein, E3 ubiquitin-ligase, *Phytophthora sojae*, oomycete, RXLR effector, plant immunity

## Abstract

*Phytophthora sojae* is an oomycete that causes stem and root rot disease in soybean. *P. sojae* delivers many RxLR effector proteins, including Avr1b, into host cells to promote infection. We show here that Avr1b interacts with the soybean U-box protein, GmPUB1-1, in yeast two-hybrid, pull down, and bimolecular fluorescence complementation (BIFC) assays. *GmPUB1-1*, and a homeologous copy *GmPUB1-2*, are induced by infection and encode 403 amino acid proteins with U-Box domains at their N-termini. Non-synonymous mutations in the Avr1b C-terminus that abolish suppression of cell death also abolished the interaction of Avr1b with GmPUB1-1, while deletion of the GmPUB1-1 C-terminus, but not the U box, abolished the interaction. BIFC experiments suggested that the GmPUB1-1-Avr1b complex is targeted to the nucleus. *In vitro* ubiquitination assays demonstrated that GmPUB1-1 possesses E3 ligase activity. Silencing of the *GmPUB1* genes in soybean cotyledons resulted in loss of recognition of Avr1b by gene products encoded by *Rps1*-b and *Rps1*-k. The recognition of Avr1k (which did not interact with GmPUB1-1) by *Rps1*-k plants was not, however, affected following *GmPUB1*-*1* silencing. Furthermore, over-expression of GmPUB1-1 in particle bombardment experiments triggered cell death suggesting that GmPUB1 may be a positive regulator of effector-triggered immunity. In a yeast two-hybrid system, GmPUB1-1 also interacted with a number of other RxLR effectors including Avr1d, while Avr1b and Avr1d interacted with a number of other infection-induced GmPUB proteins, suggesting that the pathogen uses a multiplex of interactions of RxLR effectors with GmPUB proteins to modulate host immunity.

## Introduction

*Phytophthora* root and stem rot disease caused by *Phytophthora sojae* is a destructive and the third most serious soybean disease in the U.S. (Tyler et al., 2007). In the U.S., the estimated average soybean annual yield suppression caused by this disease has been valued at $0.47 billion (Bandara et al., 2020). Losses to *P. sojae* have increased by 4-fold in the last 10 years (Wrather et al., 2001, 2003; Koenning and Wrather, 2010) as resistance genes have lost effectiveness. Soybean-*P. sojae* interactions have been extensively studied for understanding the signaling pathways involved in disease pathogenesis (Tyler, 2007, 2008).

Plants have evolved a number of strategies for combat disease-causing microorganisms (Duplan and Rivas, 2014) including effector-triggered immunity (ETI) (Gohre and Robatzek, 2008). ETI involves the direct or indirect recognition by R gene products of the presence of specific pathogen effectors. Effectors recognized by R proteins are termed avirulence (Avr) proteins. Rapid and localized cell death that limits pathogen growth, termed the hypersensitive response (HR), commonly accompanies ETI (Qutob et al., 2002; McDowell and Woffenden, 2003; Coll et al., 2011). Soybean *R* genes whose products recognize *P. sojae* Avr effectors and trigger *Phytophthora* resistance are known as *Rps* (Resistance to *P. sojae*) genes (Tyler and Gijzen, 2014). Soybean cultivars with *Rps* genes have proven to be an effective method to combat *Phytophthora* disease. One of the most durable *Rps* loci, *Rps1*-k, has been molecularly characterized. The *Rps1*-k locus carries two functional, highly similar coiled-coil nucleotide-binding and leucine-rich-repeat genes, *Rps1*-k-1 and *Rps1*-k-2 (Gao et al., 2005; Gao and Bhattacharyya, 2008). The *Rps1*-k locus enables soybean plants to recognize two genetically linked but dissimilar *P. sojae* effectors, Avr1b and Avr1k (Song et al., 2013), but it is unknown if recognition is mediated by distinct genes at the *Rps1*-k locus.

Degradation of ubiquitinated proteins through the 26S proteasome plays a major role in regulating defense responses (Goldberg, 2003; Vierstra, 2009; Marino et al., 2012; Duplan and Rivas, 2014). The formation of an isopeptide linkage between the C-terminal glycine residue of the ubiquitin peptide and the epsilon-amino groups of lysine residues of target proteins is mediated by a multi-enzyme cascade of three key enzymes: E1 (ubiquitin-activating enzyme), E2 (ubiquitin-conjugating enzyme), and E3 (ubiquitin-protein ligase) (Aravind and Koonin, 2000; Fang and Weissman, 2004). In the first step, ubiquitin is activated by E1 in an ATP-dependent manner. Then in the second step it is transferred to E2, to which it binds covalently. In the third step, E3 binds the E2-ubiquitin conjugate and the target protein and transfers the ubiquitin from E2 to a lysine residue on the target protein (Aravind and Koonin, 2000). The target specificity is determined by the E3 ubiquitin ligase. Compared to a relatively small number of E1 and E2 proteins, there are a very large number of E3 proteins with diverse specificities for E2 substrates and target proteins (Goldberg, 2003). Approximately 5% of *Arabidopsis* genes code for proteins of the ubiquitination pathway including two E1 proteins, at least 45 E2 proteins and more than 1,200 E3 proteins (Mazzucotelli et al., 2006; Yee and Goring, 2009). Depending on the structural features and mechanisms of action, E3 ligases are classified into four main sub-families: HECT (Homologous to the E6-AP Carboxy-Terminus), RING (Really Interesting New Gene), U-Box, and CRL (Cullin-RING ligases; Vierstra, 2009).

The *Arabidopsis* genome encodes 62 U-box proteins (Yee and Goring, 2009), while the soybean genome encodes more than 80 (Schmutz et al., 2010). Many of these U-Box proteins have been identified to be involved in self-incompatibility, hormone responses, biotic and abiotic stress responses (Monte et al., 2003; Stone et al., 2003; Yan et al., 2003; Cho et al., 2006, 2008; Liu et al., 2007; Samuel et al., 2008).

The importance of protein degradation in plant defenses was first established when SGT1 (Suppressor of G2 allele of SKP1), a component of the SCF complex for protein degradation, was demonstrated to be an essential component of *R*-gene mediated plant immunity (Azevedo et al., 2002). Subsequently, numerous examples of the integral role of ubiquitin-mediated degradation in the regulation of plant immunity, and its manipulation by pathogens, have been documented [reviewed in Trujillo and Shirasu (2010), Alcaide-Loridan and Jupin (2012), Marino et al. (2012), Duplan and Rivas (2014), Couto and Zipfel (2016), and Zhou and Zeng (2016)].

Plant U-Box (PUB) proteins with E3 Ub-ligase activity play an important role in regulating plant immune signaling, acting as positive or negative regulators. Positive regulators include tobacco NtCMPG1 and NtACRE276 (Gonzalez-Lamothe et al., 2006; Yang et al., 2006), the *Arabidopsis* ACRE276 homolog AtPUB17 (Yang et al., 2006; He et al., 2015), the *Arabidopsis* U-box proteins, MAC3A and MAC3B (Monaghan et al., 2009), pepper CaRING1 (Lee et al., 2011), *Arabidopsis* RGLG3 and RGLG4 (Zhang et al., 2012), and *Arabidopsis* SR1IP1 (Zhang et al., 2014). Negative regulators include rice SPL11 (Zeng et al., 2004; Shirsekar et al., 2014) and its *Arabidopsis* ortholog AtPUB13 (Li et al., 2012), the *Arabidopsis* U-box protein triplet, PUB22, PUB23, PUB24 (Trujillo et al., 2008), rice OsPUB51 (Park et al., 2012), and *Arabidopsis* ABD1 (Seo et al., 2014). In addition to U box protein genes with a demonstrated role in plant immunity, many others such as *Arabidopsis CMPG1* homologs, *AtPUB21* and *AtPUB22* are induced by infection or elicitor treatment (Navarro et al., 2004).

Effector proteins produced by pathogens and mutualists also manipulate the ubiquitin-mediated degradation pathway, some by carrying E3 ligase activity and others by manipulating plant E3 ligases (Duplan and Rivas, 2014). Examples of effectors carrying E3 ligase activity include NopM (nodulation outer protein M) of *Rhizobium* sp. strain NGR234 (Xin et al., 2012), AvrPtoB of *Pseudomonas syringae* pv. *tomato* (Martin et al., 2003; Abramovitch et al., 2006; Rosebrock et al., 2007; Mathieu et al., 2014), and AvrPtoBB728a from *Pseudomonas syringae* pv. *syringae* B728a (homologous to AvrPtoB) (Chien et al., 2013). *R. solanacearum* GALA effectors (Angot et al., 2007) and *Agrobacterium* ASK1/ASK2 proteins (Anand et al., 2012) employ an F-box domain to form an active SCF-type E3 ubiquitin ligase required for virulence. Oomycete and fungal effectors without Ub-ligase related domains can target host E3 Ub-ligases. Avirulence effector AvrPiz-t from the rice blast fungus *Magnaporthe oryzae* suppresses the ubiquitin ligase activity of the rice RING E3 ubiquitin ligase APIP6 (Park et al., 2012), Avr3a from *Phytophthora infestans* stabilizes and inhibits the function of host U-box protein CMPG1 (Bos et al., 2010), and Avr1d from *P. sojae* binds to the U-Box of GmPUB13, acting as an E2 competitor (Lin et al., 2021).

Like other oomycete and fungal pathogens, *P. sojae* delivers a large collection of effector proteins into host cells during infection to promote colonization of soybean plants (Jiang et al., 2008; Wang et al., 2011; Jiang and Tyler, 2012). Avr1b is one such effector protein, conferring enhanced virulence when overexpressed in *P. sojae* (Shan et al., 2004; Dou et al., 2008). Although there is ample evidence suggesting involvement of U-Box proteins in the defense of rice, tobacco, tomato and *Arabidopsis* against invading pathogens (Zeng et al., 2004; Yang et al., 2006; Monaghan et al., 2009; Duplan and Rivas, 2014) very little is known about soybean E3 ligase proteins, except for a recently identified RING-finger protein with polyubiquitination activity (Du et al., 2010).

In the present study, a U-Box containing protein GmPUB1-1 with E3 ligase activity was identified by screening a soybean yeast two-hybrid library using Avr1b as a bait. Further yeast two-hybrid assays revealed multiplex interactions involving additional RxLR proteins and GmPUB proteins. Silencing and over-expression experiments suggest that GmPUB1-1 may be a positive regulator of effector-triggered immunity that is guarded against Avr1b-binding by proteins encoded by genes within the *Rps1-*b and *Rps1-*k loci.

## Materials and Methods

### Plant Materials, Transformation, and Growth Conditions

Seeds of soybean cultivars Williams and Williams 82 were sown in coarse vermiculite and soaked with 3 L of water immediately after sowing. The flats were kept in a Conviron Growth Chamber (16 h photoperiod, 200 μEs, 24°C day and 20°C night temperatures) for 7 days. On day 7, cotyledons were harvested for *Agrobacterium rhizogenes*-mediated transformation. The ideal cotyledons selected for the assay were ones that were medium green and not snapping completely into two halves. The 7-day old cotyledons were gently twisted off the plants and collected in Petri dishes with moist Whatman filter paper (10 cotyledons each plate). The surfaces of individual cotyledons were sterilized with alcohol wipes (North Safety Products, Cranston, RI). A shallow circular (4 mm in diameter) wound was made about 3 mm from the petiole end of the cotyledon by a razor blade. 20 μl of *A. rhizogenes* suspension (OD 0.3) in 10 mM MgSO_4_ was added to the wound. Plates were wrapped with Parafilm and incubated in a Conviron growth chamber (16 h photoperiod, 100 μEs, 24°C day and 20°C night temperatures).

*A. rhizogenes* strain K599 was transformed with pART27 GFP empty vector or pART27 GFP carrying a *GmPUB1* inverted repeat by a freeze-thaw method. The transformants were selected on Luria-Bertani (LB) agar plates containing 50 μg/ml kanamycin and 100 μg/ml spectinomycin. Cultures for plant inoculation were prepared by inoculating 10 ml liquid LB broth (containing 50 μg/ml kanamycin and 100 μg/ml spectinomycin) with a single colony harboring the correct construct and growing the culture for two days at 28°C. Before inoculation onto the cotyledons, the cultures were centrifuged and resuspended in 10 mM MgSO_4_ for a final OD600 of 0.3.

### *P. sojae* Infection Assays on Soybean Cotyledons

*P. sojae* strains CC5C (Sandhu et al., 2004), P6497 or P7063 (Förster et al., 1994) were grown on lima bean agar or V8 agar plates for 1 week in the dark at room temperature. Six days after inoculation of soybean cotyledons with *A. rhizogenes* cells, the non-petiole ends of the cotyledons were wounded lightly with a tip of a 1 ml pipette tip. A small piece (3 mm in diameter) of lima bean or V8 agar containing *P. sojae* mycelia was cut with a cork borer and placed onto the cut surfaces of the cotyledons. The cotyledons were incubated in the growth chamber (16 h photoperiod, 100 μEs, 24°C day and 20°C night temperatures) and observed every day for recording disease development.

The disease rating scale used in cotyledonary bioassays was as described by Park et al. (2002). Numbers 1–5 indicate different sizes of disease lesions. 0, no observable symptom development from the infection point; 1, 1–10%; 2, 10–25%; 3, 25–50%; 4, 50–75%; 5, 75–100% of the cotyledon area infected. Differences in average disease lesion ratings were statistically tested using the Wilcoxon Rank Sum Test.

For detection of *GmPUB*1 transcripts, cotyledons were collected 3, 6, 9, and 12 days post-transformation with *A. rhizogenes* for the time course experiment, and at 6 days for subsequent experiments. A column section was harvested by a cork borer (5 mm in diameter) at the non-petiole end (the site used for *P. sojae* inoculation) of the cotyledon. RNA was extracted from each column section for analysis by qRT-PCR.

### Yeast Two-Hybrid Interaction Assays

Yeast two-hybrid interaction assays were carried out according to the manufacturer's protocol (Clontech Laboratories, Inc., Mountain View, CA). An unamplified prey cDNA library (>1.2 × 10^6^ colony forming units) was generated in the pB42AD vector from poly (A)^+^ RNAs prepared from Williams 82 (*Rps1*-k) etiolated hypocotyls, 2 and 4 h following inoculation with the *P. sojae* isolate CC5C (Bhattacharyya and Ward, 1986; Sandhu et al., 2004). GmPUB1-1 fragments were identified from this prey soybean cDNA library by screening with Avr1b as bait. The GmPUB1-1 fragments were recovered in the prey vector pB42AD. This interaction was confirmed by introducing Avr1b wildtype and mutant alleles into the pLexA vector into a yeast strain containing the longest fragment, GmPUB1-1^71−397^. Transformants were selected on SD/Gal/Raf/Xgal/BU salts (7 g/L Na_2_HP0_4_, 7H_2_O; 3 g/L NaH_2_PO_4_, pH 7.0)/-His/-Trp/-Ura for screening β-galactosidase (LacZ) expression and SD/Gal/Raf/-His/-Trp/-Ura/-Leu plates for leucine auxotrophic selection. Full-length *GmPUB1-1* and *GmPUB1-2* genes were PCR amplified (Forward primer: 5′-ATATGGATCCGTATGGACGAAATTGAAATCCCTG-3′; reverse primer: 5′-TATCCTCGAGTCATGGATAGGAAGATAACAAAGGTAC-3′) from genomic DNA of the soybean cultivar Williams 82 and cloned into the *Bam*HI*-Xho*I sites of the bait vector pLexA. pLexA_GmPUB1-1 and pLexA_GmPUB1-2 constructs were transformed into the yeast strain EGY48/pSH18-34 and selected on SD/-His/-Ura plates. An autoactivation assay was performed to test bait suitability. Because of the autonomous activation of *GmPUB1-1* in the LexA system, only *GmPUB1-2* was used as the bait for testing interactions of full length GmPUB proteins with Avr1b mutants. *Avr1b* genes carrying mutations in the W and Y motifs (Dou et al., 2008) and wild-type *Avr1b* were cloned into the pB42AD vector and introduced into yeast strains harboring the bait pLexA_GmPUB1-2. Double transformants were selected and screened for expression of *LEU2* and *LacZ* reporter genes. Positive interactions were visualized as blue color on SD/Gal/Raf/X-gal/BU salts/-His/-Trp/-Ura plate from activation of *LacZ* gene and also from yeast growth on minimal medium lacking leucine due to complementation for the *LEU2* gene.

For screening of GmPUB1-1, GmPUB1-2, and other PUB protein interactions with other Avr1b alleles and RxLR effectors, the effectors were cloned into pLAW10 and the PUB proteins were cloned into pLAW11. For reverse selection, the effectors were cloned into pLAW11 and the PUB proteins were cloned into pLAW10. pLAW10 and pLAW11 are Gateway™-compatible version of pGBKT7 and pGADT7, respectively (Yang et al., 2013). The genes were cloned into these vectors in a two-step process. First the genes were amplified with primers (listed in Supplementary Table 1) carrying attB sequences, then the fragments were cloned in pDONR207 *via* the Gateway™ BP reaction according to the manufacturer's protocol. Then the inserts were transferred into pLAW10 or pLAW11 using the Gateway™ LR reaction according to the manufacturer's protocol. pLAW10 constructs were introduced into yeast strain Y8930 (genotype: *MAT*α, *leu2-3,112 trp1-901 his3*Δ*200 ura3-52 gal4*Δ *gal80*Δ *GAL2-ADE2 LYS2::GAL1-HIS3 met2::GAL7-lacZ cyh2*^*R*^) using selection on –Trp medium with glucose as a carbon source. pLAW11 plasmids were introduced into Y8800 (genotype: *MAT****a****leu2-3,112 trp1-901 his3*Δ*200 ura3-52 gal4*Δ *gal80*Δ *GAL2-ADE2 LYS2::GAL1-HIS3 met2::GAL7-lacZ cyh2*^*R*^) using selection on –Leu medium with glucose as carbon source. To test for interactions, the bait- and prey-bearing strains were mated by co-inoculating 2 mL YEPD with each strain then incubating in a shaker at 240 rpm for overnight at 28°C. To select diploids, 40–80 μl of the mating cultures were plated on –Leu-Trp plates (glucose carbon course) and incubated for 40 h at 28°C. To test diploids for evidence of protein-protein interactions, single diploid colonies were inoculated into 2 mL –Leu-Trp liquid medium. After overnight incubation in a shaker with 240 rpm at 28°C, the cultures were diluted to an OD600 of 0.250 – 0.500 in a 96 well micro-titer plate. A 96-pin replicator was then used to transfer droplets from each well onto –Leu-Trp, -Leu-Trp-His, and -Leu-Trp-Ade plates. The plates were then incubated at 28°C for 3-5 days. GmPUB1-1 exhibited only very weak self-activation in pLAW10.

### *In vitro* Pull-Down Assays Using Proteins Produced by *in vitro* Transcription-Translation

To produce Avr1b and GmPUB1-1 proteins by *in vitro* transcription-translation, the full-length *Avr1b* gene in pB42AD and *GmPUB1-1* gene in pRSETA were used as the template for PCR amplification. Primers are presented in Supplementary Table 2. Forward primer with a Kozak consensus sequence and gene specific reverse primer were used in the first round of PCR. The resulting products were re-amplified with T7 promoter sequence containing forward primer and the same reverse primer. PCR products were translated using the wheat germ-based TNT^®^ T7 quick for PCR system (Promega, Madison, WI) as recommended by the manufacturer's protocol. Expressed proteins were separated by SDS-PAGE and gel blotted for western analysis to assess the size and translational efficiency of individual proteins.

For pull-down assays, 90 μl TNT^®^ reaction containing HA-Avr1b was diluted with 110 μl 1X PBS (137 mM NaCl, 2.7 mM KCl, 10 mM Na_2_HPO_4_, 2 mM KH_2_PO_4_) pH 7.4. The total volume was incubated with 6 μl anti-HA agarose slurry (Pierce Technology Corporation, Holmdel, NJ) in a Handee^TM^ Mini-Spin Column (Pierce Technology Corporation, Holmdel, NJ) for 2 h at 4°C with gentle end-over-end rotation. The column was washed three times with PBS-T (0.15 M NaCl, 25 mM Tris-HCl, pH 7.2, 0.05% Tween-20) and the agarose was resuspended in 150 μl 1X PBS. 50 μl His-GmPUB1-1 TNT^®^ reaction was added to the prepared column and incubated overnight at 4°C with end-over-end rotation. The columns were washed three times with 500 μl PBS-T buffer to remove any unbound proteins. Interacted GmPUB1 proteins were eluted with 25 μl non-reducing sample buffer (Pierce Technology Corporation, Holmdel, NJ) and boiling the slurry for 5 min. After adding 3 μl of β-mercaptoethanol, the samples were boiled again for 5 min and resolved on two 10% SDS-PAGE gels and detected with anti-His (Amersham Bioscience, PA) and anti-HA (Invitrogen, Carlsbad, CA) antibodies, respectively.

### Bimolecular Fluorescence Complementation Transient Expression Assays *in planta*

For BiFC, the Avr1b ORF sequence was cloned into pSAT1-cEYFP-C1-B to generate the C-terminal in frame fusion with cEYFP (Forward primer: 5′-GACTAAGCTTCGATGCGTCTATCTTTTGTGC-3′; reverse primer: 5′-AGTCGGATCCTCACTGGTGGTGCTGGTGGTG-3'), whereas GmPUB1-1Δ71-397 was introduced into pSAT1-nEYFP-N1 to form the N-terminal in frame fusion with nEYFP (Forward primer: 5′-GACTAGATCTCGATGCAATCTTGGTGCACCCTC-3′; reverse primer: 5′-CAGGATCCCGGGTTCCTTTGCCCTCTCCTTAG-3′). Onion lamellas were placed inside Petri dishes containing moist filter papers and then bombarded with gold particles coated with each combination of plasmids: GmPUB1-1Δ71-397-YFPN/YFPC- Avr1b fusion plasmids or YFPN/YFPC as the negative control. Onion inner layer were bombarded at 1350 PSI Helium pressure and kept in dark at 22°C post-bombardment. The EGFP signal was detected 24 h after the bombardment with a fluorescence microscope (Carl Zeiss Axiostar Plus). The microscopic field was viewed under green isothiocyanate filters. Fluorescence was detected after 24 h with a fluorescence microscope (Carl Zeiss Axiostar Plus).

### Expression and Purification of Recombinant Proteins in *E. coli*

The full-length wild type *GmPUB1-1* and mutant *GmPUB1-1* were cloned in the pRSET A (Invitrogen, Carlsbad, California) vector and used to produce 6XHis tagged fusion proteins. The mutant *GmPUB1-1* was generated by substituting three highly conserved U-box domain amino acid residues (C12A, V23A, and W39Ile) previously shown to be critical for E3 ligase activity (Gonzalez-Lamothe et al., 2006; Yang et al., 2006). Plasmids were introduced into *E. coli* strain BL21(DE3)pLysS then the cells were induced with 1 mM isopropyl-β-D-thio-galactoside (IPTG) at OD 0.6. 16 h following induction at room temperature, cells were centrifuged and protein extraction done under native conditions using Ni-NTA resin (QIAGEN, Valencia, CA) following the manufacturer's protocol. The eluted proteins were dialyzed in 1X phosphate buffered saline pH 7.4.

### *In vitro* Ubiquitination Assay

*In vitro* ubiquitination assays were conducted as described previously (Yang et al., 2006) with slight modifications. 1 μg of purified wild type and mutant His-GmPUB1 proteins, 50 ng of human recombinant E1 enzyme (Boston Biochem, Cambridge, MA), 150 ng of E2 UbcH5b (Boston Biochem, Cambridge, MA), 2 μg of HA-Ub (Boston Biochem, Cambridge, MA) were incubated in a final volume of 30 μl reaction buffer containing 50 mM Tris-HCl, pH 7.5, 10 mM MgCl_2_, 0.2 mM DTT, 3 mM ATP, 10 mM creatine phosphate and 0.1 unit of creatine phosphokinase (Sigma-Aldrich, St. Louis, MO). Reactions were incubated at 30°C for 2 h and stopped by boiling with 4X SDS-PAGE loading buffer for 5 min. 15 μl of each reaction was analyzed by electrophoresis on 10% SDS-PAGE and subjected to immunoblot analysis. Ubiquitination was detected with anti-HA antibody (Invitrogen, Carlsbad, CA) and anti-His (Amersham Bioscience, PA) antibody.

### Western Blotting

Proteins were resolved in 10% polyacrylamide gel and then transferred to a nitrocellulose membrane (Whatman Inc. Florham Park, NJ). The membrane was blocked in PBS containing 0.1% Tween 20 and 5% fat-free dry milk for 1 h at room temperature followed by three washing with PBS/0.1% Tween-20. The membrane was incubated with primary antibodies (1:1500) and then with alkaline phosphatase conjugated secondary antibodies (1:1500). Specific proteins were visualized with AP conjugate substrate kit according to the manufacturer's instructions (Bio-rad, Hercules, CA).

### RNAi Vector Construction

438 bp *GmPUB1-1* fragments were PCR amplified with two sets of primers with different flanking restriction sites (Set 1: forward primer: 5′-GCATCTCGAGGCCTTTGCTACAATGCTGCT-3′ and reverse primer: 5′-GCATGGTACCCACTCTAGCATTGGCGGAAT-3′; Set 2: forward primer: 5′-GCATGGATCCGCCTTTGCTACAATGCTGCT-3′ and reverse primer: 5′-GCATATCGATCACTCTAGCATTGGCGGAAT-3′). The PCR products were ligated into the *Xho*I-*Kpn*I and *Bam*HI-*Cla*I sites of pHANNIBAL vector (Wesley et al., 2001) as an inverted repeat. A PDK (pyruvate orthophosphate dikinase) intron was placed between the two inverted repeats to stabilize the formation of a complementary hairpin structure (Wesley et al., 2001). The fragment containing the inverted repeats was sub-cloned as a *Not*I fragment into the pART27 GFP binary vector. The pART27 GFP vector was made by inserting a 35S:GFP from p35S-GFP vector (Clontech, Mountain View, CA) into the *Sac*I site of pART27 (Wesley et al., 2001).

### RT-PCR Assays of *GmPUB1* Transcript Levels

Total RNA was extracted by TRIzol (Invitrogen, Carlsbad, California) or using a Qiagen RNeasy Plant Mini Kit (Qiagen, Valencia, CA, USA) following the manufacturers' instructions. After treatment with RNase-free DNase (Invitrogen, Carlsbad, California), 2 μg RNA was incubated with 0.5 μg oligo(dT) at 70°C for 5 min then immediately transferred to ice. After denaturation, the following reagents were added to each tube: 5 μl M-MLV 5X reaction buffer, 5 μl 2.5 mM dNTPs, 20 units of RNase inhibitor (Invitrogen, Carlsbad, California), 200 units of M-MLV reverse transcriptase (Promega, Madison, WI) and RNase-free water to make up the volume to 25 μl. Reaction tubes were incubated at 30°C for 30 min and then at 42°C for 30 min.

For RT-PCR to detect *GmPUB1* expression levels in silenced cotyledons at 3, 6, 9, and 12 days post-transformation with *A. rhizogenes*, 2 μl of 10X diluted first strand cDNA was used for specific gene amplification. *GmPUB1* primer pair (forward primer: 5′-GGCTGCATTGAAGCTCATTGTGGAGCTC-3′, reverse primer: 5′-CGCCCTTCTGCACACCCACAAAGCTGATC-3′) was used to amplify both *GmPUB1-1* and *GmPUB1-2*. The cycling parameters were 30 cycles of denaturing at 94°C for 30 s, annealing at 50°C for 30 s, and extension at 72°C for 45 s; followed by 72°C for 10 min. The digested PCR products were separated in a 2% agarose gel and visualized under UV light. The soybean *Actin* gene was used as the internal control (forward primer: 5′-CCCTCAACCCAAAGGTCAACAG-3′, reverse primer: 5′-GGAATCTCTCTGCCCCAATTGTG-3′).

Quantitative RT-PCR assays of *GmPUB1* transcript levels were conducted in a 7500 Fast Real-time PCR system (Applied Biosystems, Foster City, CA) using SYBR green. 20 ng of cDNA was used in each reaction and three technical replicates were performed. The primers used for analysis of *GmPUB1* gene expression were designed based on the overlap region at the 3'end of *GmPUB1-1* and *GmPUB1-2*. This region was not used in the RNAi construct. The sequence of the forward primer is: 5′-TCTAAGGGTCTCTCATGTGGC-3′ and the sequence of the reverse primer is 5′-GCCCCAACCTGCAACATTT-3′. *GmCYP2* gene was used as a reference gene. The primers used to amplify *GmCYP2* are: Forward primer 5′-CGGGACCAGTGTGCTTCTTCA-3′; reverse primer 5′-CCCCTCCACTACAAAGGCTCG-3′. The 2^−ΔΔCt^ method was used to quantitate relative gene expression level.

### Transcript Profiling of *GmPUB* Transcript Levels

Growth chamber-grown Williams soybean seedlings (7-day old) were inoculated in a slit in the hypocotyl with V8 broth-grown mycelia of *P. sojae* strain P6497, or hypocotyls were mock-inoculated by making an empty slit. After 12 h at 25°C in the light, the inoculated regions were harvested for RNA extraction. Total RNA was extracted from frozen and ground plant tissue using the Invitrogen Concert^TM^ Plant RNA Reagent and then pol(A)^+^ RNA was isolated using the Promega PolyATtract mRNA Isolation System III. Four independent biological replicates of 30 seedlings each were produced for mock and infected samples. The RNA samples were sequenced from the 3' end by Applied Biosystems (ABI) on their Solid™ platform. 22–27 million 25-nucleotide raw reads were obtained from each of the mock inoculated samples, and 55–66 million raw reads from each of the *P. sojae*-infected samples. Approximately 34–55% of the raw reads from each sample could be uniquely aligned to the soybean genome (Williams 82 version 1), and Reads Per Kilobase of transcript per Million mapped reads (RPKM) values were calculated for each soybean gene model for each sample using CuffLink. Differences in RPKM values between mock and infected samples were assessed using *t*-tests of log-transformed RPKM values, and using the multiple test correction of Benjamini and Hochberg (1995), using Excel software. For the *GmPUB* genes, RPKM values from closely related homeologs were averaged.

### Particle Bombardment Assays of GmPUB1-1 Over-expression

For transient expression of *GmPUB1-1* in bombardment assays, *GmPUB1-1* was amplified using the primers 5′-AGATCCCGGGGGAGCAATGAGATATGGATGAAATTGAAATCCCTGCT-3′ and 5′-GCATGGGTACCTCATGGATAGGAAGATAACAAAGGT-3′ which introduced *Xma*I and *Kpn*I sites upstream and downstream of the gene, respectively. These sites were then used to clone the gene into the bombardment vector pUC-SK (Dou et al., 2008). Bombardment assays and analysis of the resultant data were then conducted as described earlier (Kale and Tyler, 2011).

## Results

### Identification of a Soybean U-Box Protein That Interacts With Avr1b

**Figure 1:**
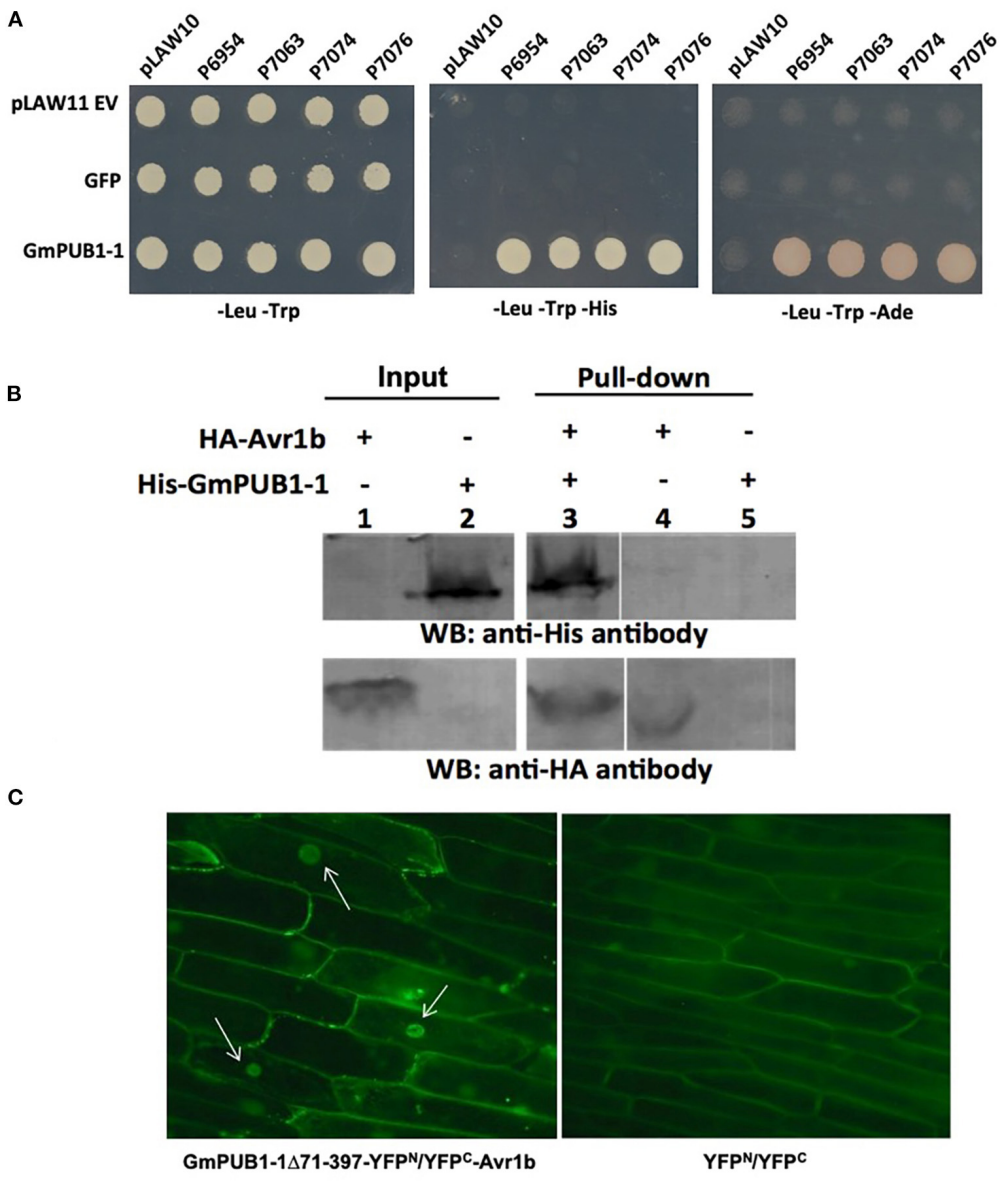
Specific interaction between GmPUB1-1 and Avr1b. **(A)** Interactions between GmPUB1-1 and four Avr1b alleles, detected by the yeast two-hybrid assay. *Avr1b* genes from *P. sojae* strains P6954 (genotype I, race 1), P7063 (genotype IV, race 6), P7074 (genotype III, race 17), or P7076 (genotype II, race 19) were cloned into bait plasmid pLAW10. *GmPUB1-1* or *GFP* genes were cloned into prey vector pLAW11. pLAW10 or pLAW11 were used as empty vector (EV) controls, respectively. Haploids carrying the bait and prey plasmids were mated, and the diploids were tested for growth in the absence of histidine (-His) or adenine (-Ade) indicative of a positive interaction. **(B)** *In vitro* interaction of Avr1b with GmPUB1 proteins. Recombinant fusion proteins HA-Avr1b and His GmPUB1-1 were expressed *via* TNT^®^ T7-quick-for-PCR DNA system (Promega, Madison, WI). Aliquots of HA-Avr1b (lane 1) or His-GmPUB1-1 (lane 2) proteins used in the pull-down assay were blotted onto membrane and probed with anti-HA and anti-His antibodies, respectively. His-GmPUB1-1 was incubated with HA-Avr1b that was immobilized onto anti-HA agarose beads overnight at 4°C. The bound proteins were eluted, resolved by SDS-PAGE and blotted onto membrane and then probed with anti-His and anti-HA antibodies, respectively (lane 3). HA-Avr1b (lane 4) or His-GmPUB1-1 (lane 5) alone were used as negative controls. Predicted sizes of the recombinant proteins are: HA-Avr1b, ~18.6 kDa; His-GmPUB1-1, ~48 kDa. **(C)** BiFC analysis showing *in planta* interaction between GmPUB1-1 and Avr1b. (Left) Nuclear fluorescence (arrows) observed from complementation of the C-terminus of YFP fused to Avr1b (Avr1b-C-EYFP) with the N-terminus of YFP fused to GmPUB1-1^71−397^ (GmPUB1-1^71−397^-N-EYFP) after bombardment into onion bulb epidermal cells. (Right) No fluorescence was detected in controls with C-EYFP and N-EYFP alone.

Screening of a yeast two-hybrid library prepared from *P. sojae* infected soybean hypocotyls using Avr1b as bait identified eight clones (Table 1). Among these eight Avr1b-interactors, three encoded portions of the same U-box protein, corresponding to gene model *Glyma*. *13G312700*, which we named *GmPUB1-1*. In the yeast two-hybrid assay, full length *GmPUB1-1* constructs produced positive interactions with Avr1b proteins encoded by *Avr1b*-*1* genes from all four genotypes of *P. sojae* (Figure 1A). In addition to the *GmPUB1-1* gene, the *Glycine max* genome contains a highly similar homeologous copy of *GmPUB1-1*, which we named *GmPUB1-2* (*Glyma.12g189000*; https://phytozome-next.jgi.doe.gov/). GmPUB1-1 shares 94% amino acid identity with GmPUB1-2. Both the *GmPUB1* genes encode proteins containing 403 amino acids with a U-Box domain located at the N-terminus. Based on the three fragments of *GmPUB1-1* recovered in the initial yeast two-hybrid screen (Table 1), the minimal region of GmPUB1-1 required for binding of Avr1b lies between residues 143 and 369 and does not include the U-box.

**Table 1:** Soybean proteins that showed *in vivo* interactions with Avr1b in yeast.

**Gene ID**	**Putative annotation**	**Gene number (amino acid represented)**	***E*** -values^*^
Avr1b_Int_1	Photosystem Ii Reaction Center PSB28 Protein, Chloroplastic-like	Glyma.13G127200 (64-179)	7e-81
Avr1b_Int_2	Protein Light-Dependent Short Hypocotyls 10-like	Glyma.13G229700 (1-157)	5e-105
Avr1b_Int_3	Photosystem II Reaction Center PSB28 Protein, Chloroplastic-like	Glyma.13G127200 (53-179)	5e-88
Avr1b_Int_4	UDP-Glucose 6-Dehydrogenase 1-like	Glyma.19G028500 (371 – 480)	2e-75
Avr1b_Int_5	E3 Ubiquitin-Protein Ligase Pub23-like Isoform X2	Glyma.13G312700 (143 – 403)	6e-166
Avr1b_Int_6	E3 Ubiquitin-Protein Ligase Pub23-like Isoform X2	Glyma.13G312700 (71 – 397)	0
Avr1b_Int_7	E3 Ubiquitin-Protein Ligase Pub23-like Isoform X2	Glyma.13G312700 (89 – 369)	0
Avr1b_Int_8	Protein Light-Dependent Short Hypocotyls 10-like	Glyma.13G229700 (1 – 163)	3e-126

* *Annotation and blast search was conducted on March 14, 2017*.

### *In vitro* Pull-Down Assay Confirms the Interaction Between Avr1b and GmPUB1-1

To verify the protein interaction observed in the yeast two-hybrid experiment, we assayed the interaction between Avr1b and GmPUB1-1 *in vitro* by conducting a pull-down assay. His-tagged GmPUB1-1 protein was incubated with HA-tagged Avr1b protein immobilized on HA beads. Unbound proteins were washed away using PBS-T buffer. Associated proteins were then eluted, separated by SDS-PAGE, and immunoblotted with anti-His and anti-HA antibodies. Figure 1B shows that His-GmPUB1-1 was pulled down by HA-Avr1b (lane 3). However, when HA-Avr1b was omitted, His-GmPUB1-1 did not appear in the pull-down fraction (lane 5), indicating the specificity of the assay. This result therefore provides *in vitro* confirmation of the interaction between GmPUB1-1 and Avr1b.

### Complexes of Avr1b and GmPUB1-1 Become Predominantly Localized to the Nuclei *in planta*

Bimolecular fluorescence complementation (BiFC) assays were conducted to examine the interaction of Avr1b and GmPUB1-1 *in planta*. Onion cells were bombarded with two plasmids carrying either Avr1b or GmPUB1-1 fused to split yellow fluorescence protein; i.e., in one construct Avr1b was fused to the C-terminal half of YFP and in the other construct, GmPUB1-1^71−397^ (lacking the entire U-box domain) was fused to the N-terminal half of YFP. Assuming Avr1b and GmPUB1-1^71−397^ interacted *in planta*, the interaction would bring the two halves of YFP into close proximity to form a functional fluorophore. Empty vector combinations (half of YFP not fused to an interaction partner) were used as the negative control. As shown in Figure 1C, bright fluorescence was detected in nuclei with weaker fluorescence in the cytoplasm, consistent with Avr1b interacting with GmPUB1-1 in plant cells, and with the complex being predominantly targeted to nuclei. No fluorescence was detected in the negative control (Figure 1C).

### Mutations in the C-Terminal W-Y Domain of Avr1b Result in Loss of Interaction With GmPUBs

**Figure 2:**
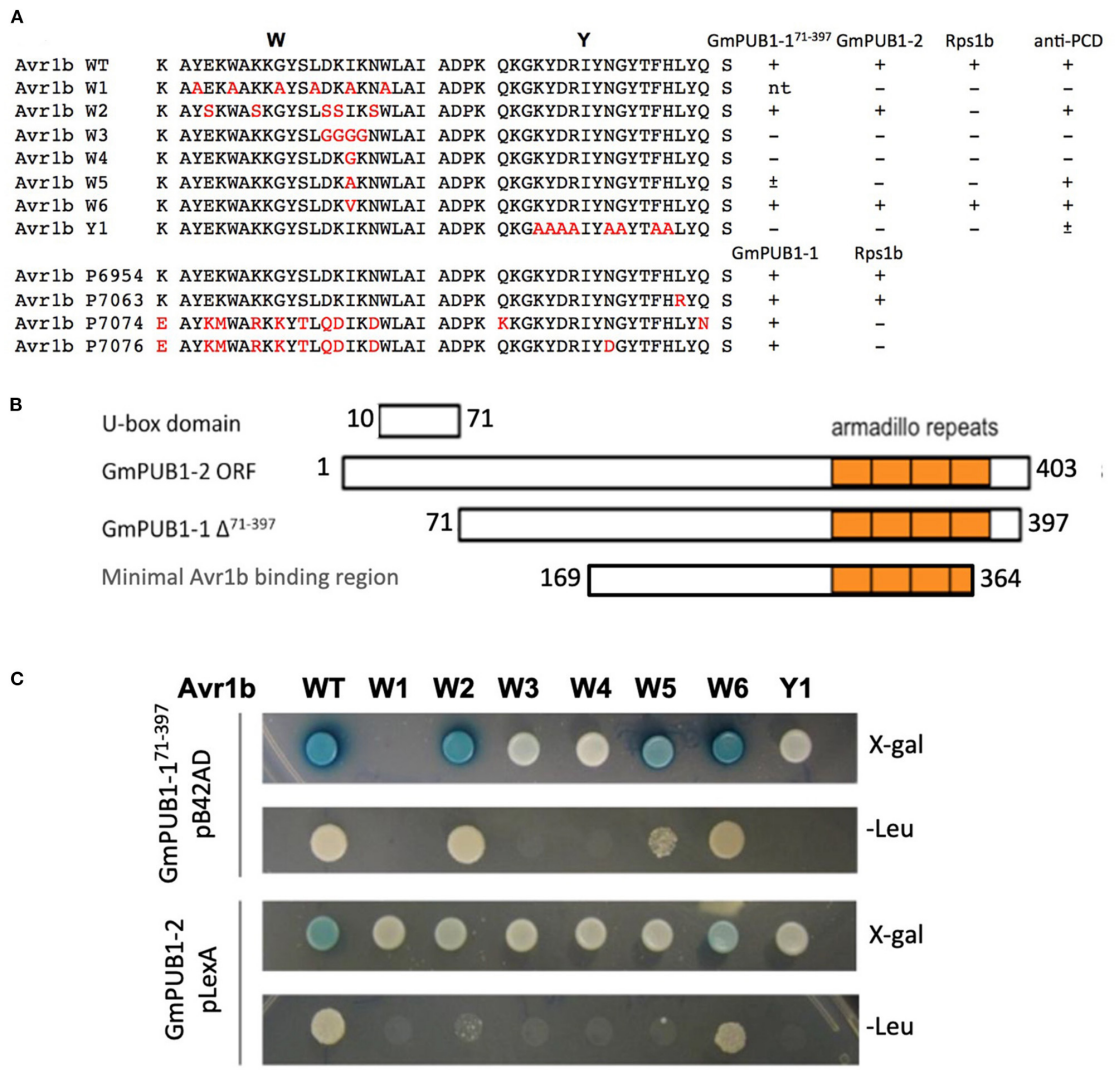
Interactions of GmPUB1s with Avr1b and mutant Avr1b proteins. **(A)** Sequences of W and Y motifs of Avr1b, Avr1b mutants, Avr1b variants and their interaction with GmPUB1s and PCD responses in an *Rps1*-b containing line. Sequence differences are in red. +, interaction; -, no interaction; ±, partially active; n.t., not tested. Anti-PCD activity was cited from Dou et al. (2008), indicating the ability of Avr1b to suppress pro-apoptopic protein BAX-induced programmed cell death (PCD) in soybean leaves containing *Rps1-*b. **(B)** Schematic representation of protein fragment of GmPUB1s used in yeast two-hybrid assay, and the minimal region required for Avr1b binding, based on the fragment of GmPUB1-1 recovered in the original two-hybrid screen (Table 1). **(C)** Yeast two-hybrid assays for *in vivo* interactions of GmPUB1 with Avr1b and mutant Avr1b proteins. Yeast strain EGY48/pSH18-34 containing combinations of truncated GmPUB1-1 in the prey vector pB42AD and C-terminal mutated forms of Avr1b in the bait vector pLexA, or full length GmPUB1-2 in the bait vector pLexA and Avr1b mutants in the prey vector pB42AD were grown on SD/Gal/Raf/X-gal/BU/-His/-Trp/-Ura plate and SD/Gal/Raf/-His/-Trp/- Ura/-Leu plate for 2 days to allow detection of *LacZ* and *Leu2* gene expression respectively. Blue color in X-gal containing plate and colony growth on minimum medium lacking leucine suggest the presence of protein-protein interactions.

More than half of the *Phytophthora* effector proteins share conserved W and Y motifs in their C-terminal domains, which form a conserved structural fold (Win et al., 2012b). Different amino acid substitutions in the C-terminal W and Y motifs of Avr1b abolish recognition of the protein by *Rps1*-b plants or the ability of Avr1b to suppress cell death in soybean lines lacking *Rps1*-b (Shan et al., 2004; Dou et al., 2008). To test whether these two motifs might also be involved in interaction with GmPUB1 proteins, a yeast two-hybrid assay was conducted to test the interaction of the mutant proteins with GmPUB1-1. Constructs encoding Avr1b point mutants in the W and Y motifs (Figure 2A) were cloned into the “bait” vector pLexA. For the “prey” plasmid, we used the C-terminal fragment of GmPUB1-1 (GmPUB1-1^71−397^ lacking the U box domain) that was recovered in the original yeast two-hybrid screen (Figure 2B). The “bait” and “prey” plasmids were introduced into the yeast strain EGY48/pSH18-34. This yeast strain has two reporter genes, *LEU2* and *LacZ*. Protein interaction results in β-galactosidase (LacZ) activity and growth in medium lacking leucine (Leu2). Avr1b mutants W3, W4, and Y1 did not interact with GmPUB1- 1^71−397^ (Figure 2C); whereas the wild type and mutants W2, W5 (weakly) and W6 did interact. In order to examine interactions between full-length GmPUB1s and Avr1b, we cloned *GmPUB1-1* into the bait vector, pLexA. However, GmPUB1-1 displayed strong self-activation, so reliable results could not be obtained. GmPUB1-2 did not display self-activation; and therefore, considering the high identity (94%) between GmPUB1-1 and GmPUB1-2, we tested the interactions of full length GmPUB1-2 with the Avr1b wild type and mutants. GmPUB1-2 reproducibly interacted with Avr1b and some of its mutants, albeit more weakly than GmPUB1-1 (Figure 2C). Avr1b wild type and mutant W6 preserved some ability to interact with GmPUB1-2 but interaction with W2 was especially weak, and no interaction with W5 could be detected (Figure 2C). In summary, mutations such as W3 and W4 that abolish the ability of Avr1b to suppress cell death also abolished the ability to interact with GmPUB1-1; whereas mutations such as W2 that abolish recognition of Avr1b by Rps1-b, but not cell death suppression, did not abolish the GmPUB1-1 interaction. Mutants W5 and Y1 showed partial departure from this pattern: W5 abolished cell death suppression but only weakened the interaction with GmPUB1-1, while Y1 abolished the interaction with GmPUB1-1 but only weakened cell death suppression. All mutations that abolished the interaction also abolished recognition by *Rps1-*b-containing soybean lines. However, some mutations that abolished recognition by *Rps1*-b-containing lines did not abolish the interaction with GmPUB1-1. These results suggest that the interaction is necessary but not sufficient for recognition by *Rps1-*b plants. Overall, the results from the mutation analysis support that the interaction between Avr1b and GmPUB1-1 is relevant to the functioning of Avr1b inside soybean cells.

### GmPUB1-1 Has Ubiquitin-E3 Ligase Activity

**Figure 3:**
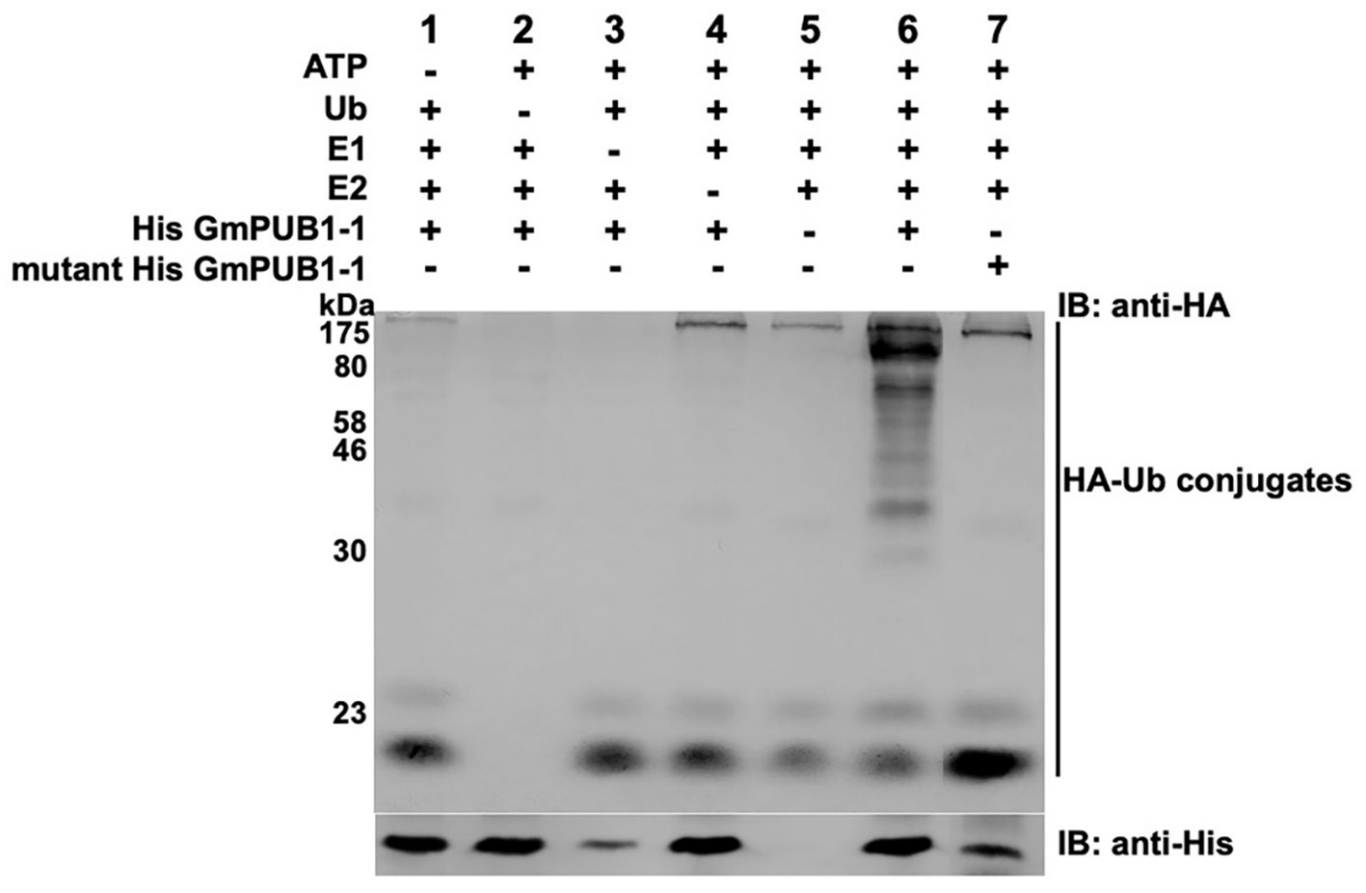
E3 Ubiquitin ligase activity of GmPUB1-1. *In vitro* ubiquitination assay with *E. coli* expressed His-GmPUB1-1 was performed in presence of combination of components as listed on top of the Figure. As a negative control for the E3 activity, a mutated version of His-GmPUB1-1 was used (three conserved amino acid residues Cys-12, Val23 and Trp-39 mutated to Ala, Ile and Ala respectively, in U-box domain). The immunoblots were probed with anti-HA antibody **(top)** to detect ubiquitinated *E. coli* proteins. Anti-His antibody **(bottom)** was used to detect His-GmPUB1-1 (~ 48 kDa) and its mutated version. Numbers on the left are molecular weight markers in kDa.

To determine whether GmPUB1-1 protein is a functional E3 ubiquitin ligase, full-length wild type GmPUB1-1 protein was expressed in *E. coli* as a fusion protein with a His tag. As a negative control, we also expressed mutant GmPUB1-1m3 protein, with three substitution mutations of highly conserved U-box domain amino acids, Cys-12 to Ala, Val-23 to Ile, and Trp-39 to Ala. The purified proteins were used for *in vitro* ubiquitination assays. His-tagged GmPUB1-1 and GmPUB1-1m3 were incubated with or without HA-Ub, ATP, E1 protein and E2 protein for 2 hours followed by western blot analysis with anti-HA and anti-His antibodies. In the presence of E1, E2, Ub, ATP and GmPUB1-1, ubiquitination of *E. coli* proteins (lane 6 in Figure 3) was detected with a monoclonal anti-HA antibody. No clear protein ubiquitination was detected in the absence of ATP, Ub, E1, E2 or E3 (lanes 1–5, Figure 3). Mutation of the conserved U-Box domain residues in GmPUB1-1m3 abolished the E3 activity (lane 7, Figure 3; Gonzalez-Lamothe et al., 2006; Yang et al., 2006). These results indicate that the U-Box domain of GmPUB1-1 protein possesses E3 ligase activity.

### *GmPUB1* Silencing Compromises *Rps1-*b- and *Rps1-*k-Mediated *P. sojae* Resistance Triggered by Avr1b

**Figure 4:**
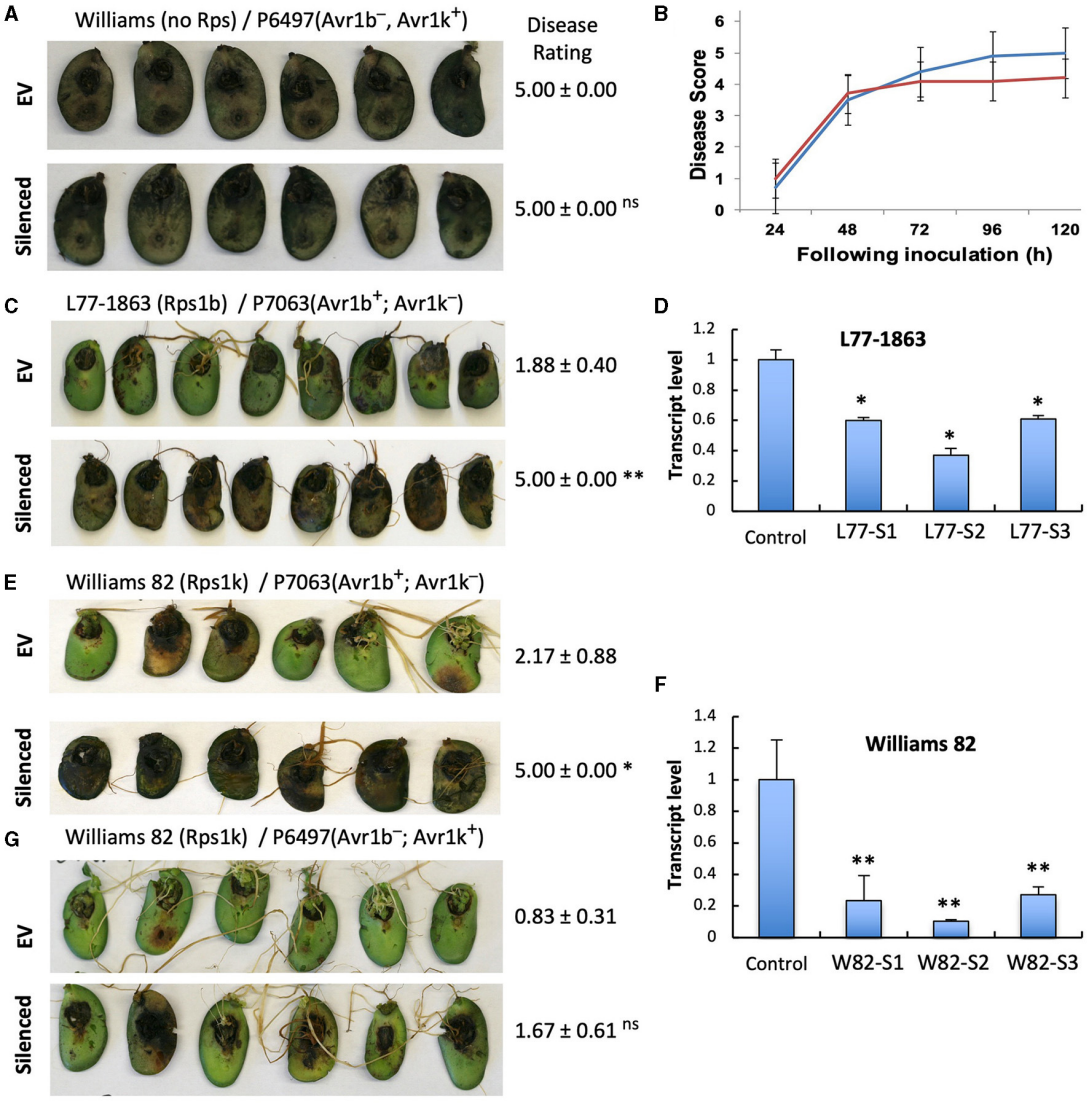
Silencing of *GmPUB1* genes resulted in specific loss of Avr1b recognition by products of the *Rps1*-b and *Rps1*-k loci. **(A,C,E,G)** Soybean cotyledons carrying *Rps1*-b **(C)**, *Rps1*-k **(E,G)**, or no *Rps* gene **(A)** were inoculated with *A. rhizogenes* harboring the *GmPUB1-1*:RNAi vector or pART27GFP empty vector. Six days later, the cotyledons were inoculated with mycelia of *P. sojae* strains P6497 **(A,G)** or P7063 **(C,E)**. Cotyledons were photographed 3 days after *P. sojae* inoculation and the disease rating scored as described in the Methods. True disease lesions showed diffuse brown margins. HR lesions were smaller and showed sharp black margins. Disease rating means and standard errors are shown. Significance of the differences between empty vector (EV) and silenced cotyledons were determined by the Wilcoxon Rank Sum Test; ^*^p < 0.05, ^**^p < 0.01, ns = not significant *p* > 0.05. *GmPUB1-1* and *GmPUB1-2* sequences are near-identical and expected to be co-silenced. **(B)** Disease development over time following inoculation with *P. sojae* CC5C. Means and standard errors were calculated from 20 infected cotyledons. Red line represents the *GmPUB1* silenced cotyledons and blue line represents the empty vector transformed cotyledons. **(D,F)** Transcript levels of *GmPUB1* (*GmPUB1-1* and *GmPUB1-2* combined) were measured by qRT-PCR in three individual soybean cotyledons of L77-1863 **(D)** or Williams 82 **(F)** inoculated with *A. rhizogenes* harboring the *GmPUB1-1*:RNAi vector. Error bars represent standard errors from three technical replicates. ^*^ and ^**^ indicate *p* < 0.01 and 0.001, respectively, for the difference with the control.

**Figure 5:**
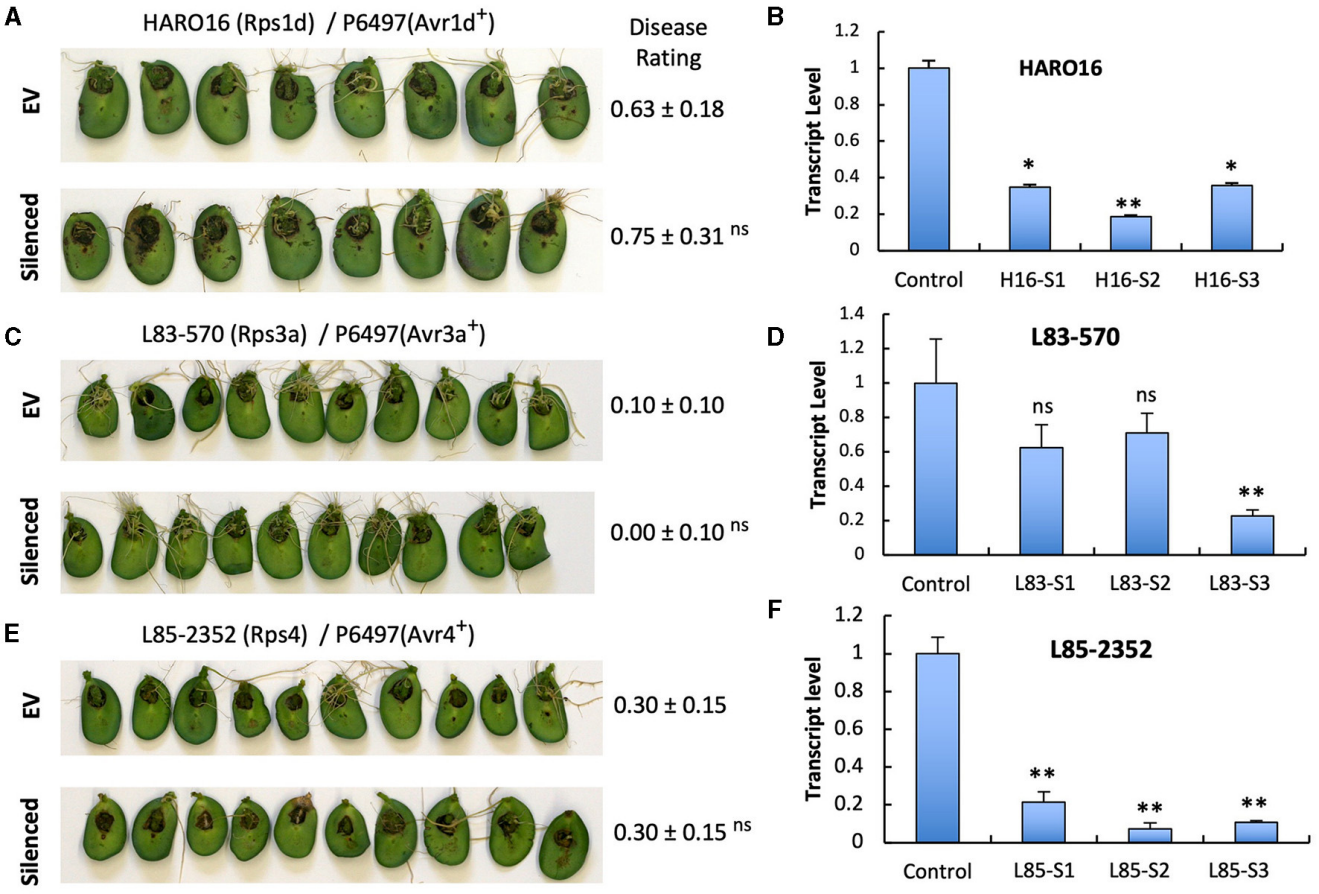
Silencing of *GmPUB1* genes does not affect effector recognition by products of the *Rps1*-d, *Rps3*-a, and *Rps4* genes. **(A,C,E)** Soybean cotyledons carrying *Rps1*-a **(A)**, *Rps3*-a **(C)**, or *Rps4* **(E)** were inoculated with *A. rhizogenes* harboring the *GmPUB1-1*:RNAi vector or pART27GFP empty vector. Six days later, the cotyledons were inoculated with mycelia of *P. sojae* strains P6497. Cotyledons were photographed 3 days after *P. sojae* inoculation and disease levels were assessed as in Figure 4. **(B,D,F)** Transcript levels of *GmPUB1* (*GmPUB1-1* and *GmPUB1-2* combined) were measured by qRT-PCR in three individual soybean cotyledons of HARO16 **(B)**, L83-570 **(D)**, or L85-2352 **(F)** inoculated with *A. rhizogenes* harboring the *GmPUB1*-1:RNAi vector. Error bars represent standard errors from three technical replicates. ns, ^*^ or ^**^ indicate *p* > 0.05, *p* < 0.01 or *p* < 0.001, respectively, for the difference with the control.

To investigate if the two *GmPUB1* genes are required for *P. sojae* resistance in soybean, we silenced *GmPUB*1-1 and *GmPUB1-2* jointly using a dsRNA construct in an *Agrobacterium rhizogenes* cotyledon assay developed by Subramanian et al. (2005). It was previously reported that silencing of the isoflavone synthase (*IFS*) gene occurred systemically in soybean cotyledons distal to *A. rhizogenes*-inoculation sites, and the most effective silencing period was between 5 and 7 days following transformation (Subramanian et al., 2005). We produced the *GmPUB1* RNAi constructs by cloning a 438 bp DNA fragment of the *GmPUB1-1* gene as inverted repeats separated by an intron in the pART27 vector (Wesley et al., 2001). The *GmPUB1-1* 438 bp fragment was highly similar to *GmPUB1-2*, and was expected to silence both genes. Soybean cotyledons were transformed with *A. rhizogenes* strain K599 carrying the empty vector pART27 *GFP* or the *GmPUB1-1* RNAi construct. Cotyledon tissues distal to the *A. rhizogenes*-inoculation sites were collected 3, 6, 9, 12 days following transformation. RT-PCR assays revealed that *GmPUB1* silencing was maximal at around 6 days (Supplementary Figure 1). To determine the effect of *GmPUB1* silencing on resistance to *P. sojae*, on day 6 an agar pulp containing *P. sojae* mycelia was placed on wounded sites distal to the *A. rhizogenes*-inoculation sites and symptoms were recorded every 24 h period following infection. When susceptible cultivar Williams was inoculated with *P. sojae* strain P6497 (race 2), which does not express Avr1b, there was no obvious difference in the susceptibility of the cotyledons in which *GmPUB1* genes were silenced (Figures 4A,B). Dark brown, water-soaked lesions spread to the entire cotyledon at the same rate in each case. Avr1b is recognized by the products of two *Rps* genes, *Rps1*-b, and *Rps1*-k. When cultivars L77-1863 or Williams 82 containing *Rps1*-b or *Rps1*-k respectively in the Williams background were used, silencing of the *GmPUB1* genes in both genotypes caused loss of resistance to *P. sojae* strain P7063 (race 6), which expresses Avr1b (Figures 4C–F). However, the effect of silencing was specific to recognition of Avr1b; when Williams 82 (*Rps1-*k) was inoculated with *P. sojae* P6497, which expresses a different effector recognized by *Rps1*-k plants, namely Avr1k, silencing of *GmPUB1* genes did not result in loss of resistance (Figures 4F,G). Furthermore, when P6497 was used to inoculate soybean cultivars containing *Rps1*-d, *Rps3*-a, or *Rps4* and silenced for *GmPUB1* genes, no loss of recognition was observed (Figure 5). Thus, silencing of *GmPUB1* genes only abolished resistance triggered by Avr1b.

To determine if GmPUB1-1 may be a positive or negative regulator of cell death, a double-barrel-bombardment transient assay (Kale and Tyler, 2011) was conducted using the reporter gene GUS. By using a control bombardment side-by-side with the test (i.e., *GmPUB1-1*) bombardment, this assay can directly measure cell death triggered by a gene of interest. It was observed that transient expression of GmPUB1-1 caused significant (*p* < 0.001) cell death (Supplementary Figure 2) suggesting that GmPUB1-1 might be a positive regulator of cell death.

### GmPUB Family Proteins Interact With Multiple *P. sojae* RxLR Effectors

**Figure 6:**
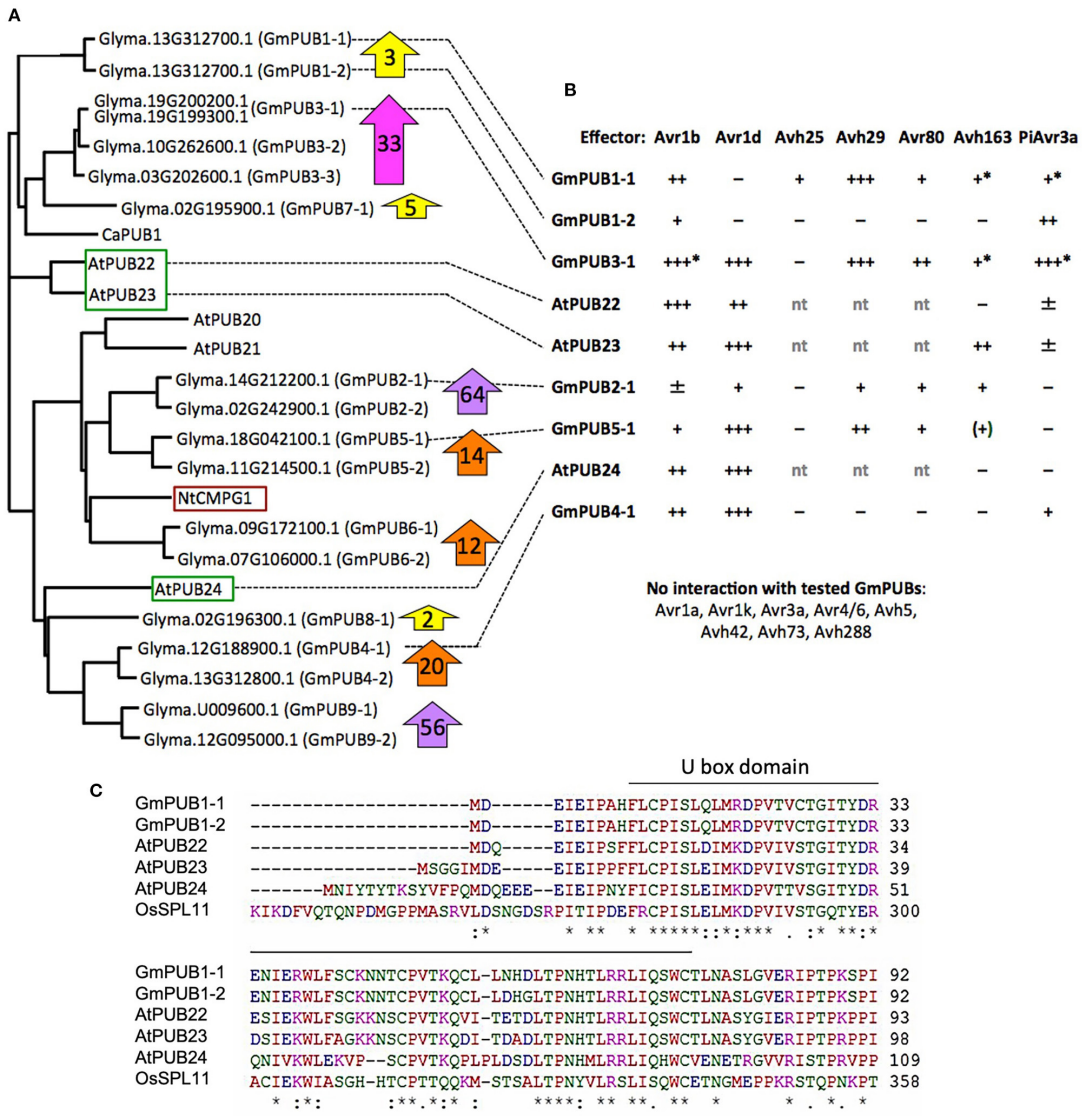
Interaction of GmPUB1-1 and paralogs with multiple RxLR effectors. **(A)** Distance tree of selected soybean and *Arabidopsis* PUBs, including known regulators of defense (red box, positive regulator; green box, negative regulators). Glyma, soybean (Williams 82 assembly v2 annotation 1); At, *Arabidopsis*; Nt, *Nicotiana tabacum*; Ca, *Capsicum annuum*. ClustalW was used for the sequence alignment and Phylip was used to create an unrooted UPGMA tree. Block arrows indicate fold change in soybean transcript levels following *P. sojae* infection (Supplementary Table 3). Levels for closely related genes are combined. **(B)** Interactions of PUBs shown in **(A)** with RxLR effectors. Most assays were done with pLAW10 (bait vector) and pLAW11 (prey vector). +++, strong interaction (growth on –Ade and –His); ++, medium (growth only on –His); +, weak (limited growth on –His); ±, very weak (micro-colonies on –His); –, negative. nt, not tested; ^*^confirmed with bait-prey swap; (+) interaction observed only with effector as bait, but not with effector as prey. **(C)** Comparison of selected U-Box domain sequences from soybean, *Arabidopsis* and rice. Amino acids with a single color are clusters of amino acids with similar physiochemical properties. Amino acids that are identical in all six proteins are marked with asterisk. Conserved and semi- conserved substitutions are marked with colon and dot.

Phylogenetic analysis (Figure 6A) revealed that GmPUB1-1 and GmPUB1-2 share close similarity with *Arabidopsis* U-box proteins, AtPUB22, AtPUB23, and AtPUB24 (Trujillo et al., 2008). GmPUB1-1 and GmPUB1-2 belong to a clade of U box proteins containing 17 GmPUB proteins (including seven pairs of homeologs), as well as U box proteins from *Arabidopsis* (AtPUB20-24), *Nicotiana* (CMPG1) and *Capsicum* (CaPUB1) (Figure 6A). Many of the *GmPUB* genes are strongly up-regulated during *P. sojae* infection (Figure 6A; Supplementary Table 3). To determine if Avr1b could interact with other U-box proteins in the same clade as GmPUB1-1, and determine if other RxLR effectors could interact with GmPUB1-1, we conducted the yeast two-hybrid assay to screen 15 RxLR effectors (including Avr1b and six other Avr effectors) against 6 representative GmPUB proteins (including GmPUB1-1 and GmPUB1-2) as well as the three AtPUB proteins most closely related to GmPUB1-1.

Five effectors (Avr1b, Avr1d, Avh29, Avh80, and Avh163) showed medium to strong interactions in the yeast two-hybrid assay with different subsets of GmPUBs 1, 3, 4, and 5, as well as with AtPUBs 22, 23, and 24 (Figure 6B; Supplementary Figure 3). Upon alignment of the protein sequences, the U-box domains of the GmPUB1 proteins were found to share 77 and 73% identity with the *Arabidopsis* PUB22 and PUB23 proteins, while PUB24 showed 66% identity (Figure 6C). Avr1b interacted most strongly with GmPUB3-1 and AtPUB22, while interactions with GmPUB4-1, AtPUB23, and AtPUB24 were comparable to its somewhat weaker interaction with GmPUB1-1. Avr1d interacted strongly with GmPUB3-1, GmPUB4-1, GmPUB5-1, AtPUB23, and AtPUB24, but not at all with GmPUB1-1 or GmPUB1-2. Avh29 interacted equally strongly with GmPUB1-1 and GmPUB3-1. *P. infestans* effector PiAvr3a, which is a homolog of Avr1b that interacts with *Nicotiana* CMPG1 (Bos et al., 2010), also showed interactions with several GmPUBs but not AtPUBs (Figure 6B). GmPUB3-1 interacted strongly with all of the RxLR effectors, except Avh80 which showed a moderate interaction. GmPUB1-2 showed only a weak interaction with Avr1b and a moderate interaction with PiAvr3a. These results suggest that GmPUBs are targeted by multiple *P. sojae* RxLR effectors, presumably to interfere with regulation of defense responses.

## Discussion

In this study, cDNA clones encoding an Avr1b-interacting protein fragment, GmPUB1-1^71−397^, were identified by screening a yeast two-hybrid library generated from *P. sojae*-infected etiolated soybean hypocotyls. The gene encoding the interactor, named *GmPUB1-1*, corresponded to soybean gene *Glyma.13G312700*. The homeologous gene *GmPUB1-2* (*Glyma.12g189000*) encoded a protein with 94% identity to GmPUB1-1, which also interacted with Avr1b in the yeast two-hybrid assay, albeit more weakly (Figure 1A). Pull-down (Figure 1B) and BIFC (Figure 1C) assays supported that GmPUB1-1 is most likely a bona fide target of the *P. sojae* effector protein, Avr1b.

Full length GmPUB1-1 and GmPUB1-2 interact with Avr1b, but so does the original interacting cDNA, GmPUB1-1^71−397^, which entirely lacks the U-box. This suggests that the N-terminal U-Box region is not required for the interactions of GmPUB1-1 or GmPUB1-2 with Avr1b. This contrasts with the binding of *P. sojae* Avr1d to the U-Box of GmPUB13 (Lin et al., 2021). U-Box domains are known to bind E2 (ubiquitin conjugating enzyme) and to be responsible for E3 ligase activity, while C-terminal domains are known to bind substrates targeted for ubiquitination (Azevedo et al., 2001). The weaker binding by GmPUB1-2 compared to GmPUB1-1, despite the 94% amino acid sequence identity between the two, suggests that the some of the polymorphic residues may contribute to the Avr1b interaction.

More than half of all oomycete effectors, including Avr1b, contain the conserved W and Y C-terminal motifs (Jiang et al., 2008) that comprise a common structural fold (Win et al., 2012b). Investigation of the Avr1b mutants (Figure 3) suggested that both the W and Y domains were required for interaction of Avr1b with GmPUBs. Furthermore, the same domains of Avr1b are most likely involved in both the interaction with GmPUB proteins and suppression of programmed cell death (Dou et al., 2008). Mutations in the W and Y motifs of Avr1b abolish Avr1b's ability to suppress cell death triggered by BAX (Dou et al., 2008) (Figure 2A). The pattern of responses to different W and Y motif mutations closely matched the pattern observed for recognition for suppression of BAX-triggered PCD by Avr1b (Figure 2A). The similarity of the yeast two-hybrid interaction patterns and anti-PCD activities of the Avr1b mutants supports that (i) the interactions of the GmPUBs with Avr1b are most likely biologically relevant and (ii) one or more of the GmPUBs may mediate the ability of Avr1b to suppress defense-related PCD.

Another study (Bos et al., 2010) has also reported that the N-terminal signal peptide and RxLR domain of Avr3a effector from *P. infestans* are not needed and that C-terminal domain with conserved W and Y motifs is sufficient for its interaction with the plant U-Box protein CMPG1 (R protein) and suppression of cell death. It was also reported that W and Y domains of effector proteins are important for interactions with R proteins and effector-related activities (Win et al., 2007). Lin et al. (2021) showed that *P. sojae* effector Avr1d binds to the U-box domain of GmPUB13 *via* its W, Y, and L domains. By solving the crystal structure of the Avr1d-GmPUB13 PUB domain complex Lin et al. (2021) showed that Avr1d occupies the binding site for E2 ubiquitin conjugating enzyme on GmPUB13.

Our BIFC study suggested that the complex of Avr1b and GmPUB1-1^71−397^ mostly localizes to nuclei (Figure 1C). Nuclear localization is required for Avr1b's avirulence activity mediated by *Rps1*-b and for suppression of effector-triggered PCD (Dou et al., 2008). Our data do not indicate whether Avr1b and GmPUB1-1 form a complex before moving into the nuclei or whether Avr1b moves into the nucleus before interacting with GmPUB1-1 located in the nuclei.

We confirmed that the U-box domain of GmPUB1-1 possesses ubiquitination activity and presumably therefore is involved in the 26S proteasome degradation pathway. In our assays, GmPUB1-1 was able to conjugate ubiquitin moieties to *E. coli* proteins. Furthermore, mutation of the conserved Cys-12, Val-23, and Trp-39 residues within the U-Box domain completely abolished ubiquitin ligase activity (Figure 3). Similar *in vitro* ubiquitin E3 ligase activity has been observed in a number of other U-Box proteins (Jiang et al., 2001; Murata et al., 2001; Ohi et al., 2005). Mutations in highly conserved amino acid residues in the U-box central core domain, such as valine, can abolish the E3 ligase activity of U-Box proteins by disrupting their tertiary structures (Ohi et al., 2003).

Several U-box proteins have been identified as positive or negative regulators of plant defense. In rice, mutations in the U-Box Spl11 protein result in spontaneous cell death and enhanced disease resistance (Zeng et al., 2004). In *N. benthamiana* and tomato, the U-box protein CMPG1 is a positive regulator required for the full hypersensitive response (Gonzalez-Lamothe et al., 2006). Also, CMPG1 is targeted by the *P. infestans* effector Avr3a (Bos et al., 2010). Another positive regulator is AtPUB17 and its *Nicotiana* ortholog ACRE276 (Yang et al., 2006). The rice pathogen *Magnaporthe oryzae* effector AvrPi-zt targets a rice RING E3 ligase (Park et al., 2012).

In *Arabidopsis*, AtPUB13 (Li et al., 2012) and a closely related trio of U-box proteins, AtPUB22, AtPUB23 and AtPUB24 (Trujillo et al., 2008) negatively regulate resistance against *H. arabidopsidis* and the bacterial pathogen *Pseudomonas syringae*; over-expression results in increased susceptibility, while the triple mutant for the U-box trio is more resistant (Trujillo et al., 2008). Using a yeast two-hybrid system, a subunit of the exocyst complex, Exo70B2, has been shown to be a substrate of AtPUB22 that mediates its ubiquitination and degradation *via* the 26S proteasome (Stegmann et al., 2012). Degradation of Exo70B2 leads to reduction in the transport of toxic chemicals such as ROS to the infection sites and attenuation of PAMP-triggered immunity responses.

The phylogenetic tree (Figure 6A) placed the GmPUB1 proteins close to the triplet of *Arabidopsis* U-Box E3 ligases PUB22, PUB23, PUB24, which are negative regulators of PAMP-triggered immunity (Trujillo et al., 2008). However, in our study it was observed that over-expression of GmPUB1-1 causes cell death (Supplementary Figure 2) suggesting that GmPUB1-1 might be a positive regulator of cell death.

**Figure 7:**
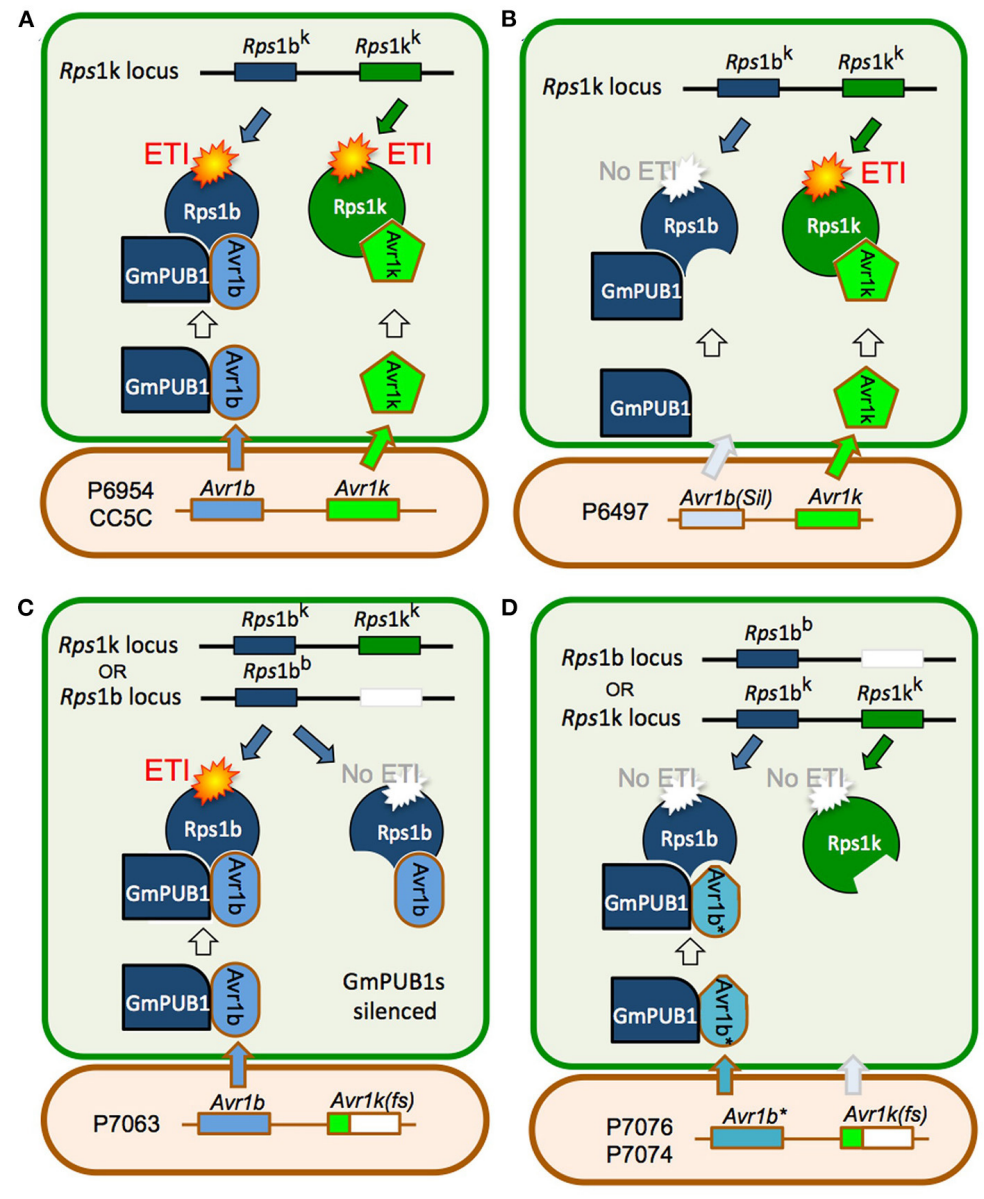
Model for the role of GmPUB1 proteins in recognition of Avr1b by plants carrying *Rps1*-b and *Rps1*-k. **(A)** *P. sojae* isolates of race 1 such as P6954 (genotype I) and CC5C, produce two completely different RxLR effectors, Avr1b and Avr1k, encoded by closely linked genes. The *Rps1*-k locus is proposed to encode two distinct proteins, Rps1-b^k^ and Rps1-k^k^, responsible for recognition of Avr1b and Avr1k, respectively, triggering ETI. Recognition of Avr1b, but not Avr1k, requires the presence of GmPUB1 (either to form a physical complex as diagrammed, or to modify Avr1b). **(B)** When *Avr1b* is silenced, either experimentally or, naturally as in the case of *P. sojae* strain P6497 (race 2, genotype I), recognition by plants containing *Rps1*-k still occurs (*via* Rps1-k^k^), but plants containing *Rps1*-b cannot recognize the *P. sojae* strain (not diagrammed). **(C)** The soybean locus *Rps1*-b is proposed to encode an Rps1-b^b^ protein (functionally identical to Rps1-b^k^) but no Rps1-k^k^ protein. When the soybean *GmPUB1* genes are silenced, Avr1b cannot be recognized by plants containing either *Rps1*-b or *Rps1*-k. If the *P. sojae* strain lacks Avr1k, as in the case of P7063 (race 6, genotype IV), silencing of *GmPUB1* genes makes both *Rps1*-b and *Rps1*-k plants susceptible. **(D)** Some *P. sojae* strains such as P7076 (race 19, genotype II) or P7074 (race 17, genotype III) produce a variant Avr1b^*^ protein that can bind to GmPUB1-1 but cannot be recognized either by plants carrying *Rps1*-b or *Rps1*-k. Since these strains also lack *Avr1k*, they can successfully infect plants carrying either *Rps1*-b or *Rps1*-k irrespective of the presence of GmPUB1 proteins.

Joint silencing of *GmPUB1-1* and *GmPUB1-2* in soybean cotyledons resulted in loss of the *P. sojae* resistance normally mediated by the recognition of Avr1b by the products of genes at the *Rps1-*b and *Rps1-*k loci. On the other hand, *P. sojae* resistance mediated by the recognition of Avr1k by the products of genes at the *Rps1-*k locus was not affected by silencing of the *GmPUB1* genes. Avr1k, which has a completely different amino acid sequence than Avr1b, did not interact in the yeast two-hybrid assay with GmPUB1-1, GmPUB1-2, or any of the other tested GmPUB proteins. These results suggest that at least one of the GmPUB1 proteins is essential for recognition of Avr1b by proteins encoded by genes at the *Rps1*-b and *Rps1-*k loci. Since recognition of Avr1k was unaffected by the silencing of the *GmPUB1* genes, and Avr1k has a different amino acid sequence than Avr1b, we speculate (Figure 7) that genes at the *Rps1-*k locus encode at least two different proteins, one able to recognize Avr1b dependent on GmPUB1 (Rps1-b^k^) and one able to recognize Avr1k independent of GmPUB1 (Rps1-k^k^). We further speculate that the *Rps1*-b locus, which is allelic to the *Rps1*-k locus, encodes a protein similar or identical to Rps1-b^k^, namely Rps1-b^b^. The *Rps1-*b locus presumably does not contain a functional allele encoding a protein similar or identical to the Rps1-k^k^ protein. The *Rps1*-k locus indeed contains two highly similar functional CC-NB-LRR type *Phytophthora* resistance genes (Gao et al., 2005; Gao and Bhattacharyya, 2008), though it is currently unknown if they differentially recognize Avr1b and Avr1k.

One possible mechanistic explanation for the requirement for GmPUB1 for recognition of Avr1b is that the Rps1-b^b^ and Rps1-b^k^ proteins guard the GmPUB1 proteins, and that binding of GmPUB1 proteins by Avr1b disrupts the binding of GmPUB1 to the Rps1b proteins, triggering defense signaling by the Rps1b proteins. However, several lines of evidence suggest that this conventional guardee scenario is not correct. First, GmPUB1-1 and GmPUB1-2 bound to all alleles of Avr1b in yeast two-hybrid assays, even alleles (from P7076 and P7074) that do not trigger *Rps1*-b-mediated resistance. Second, in other gene-for-gene systems, Avr effector proteins that interact directly with the cognate R protein, such as AvrPi-ta (Jia et al., 2000), AvrL567 (Dodds et al., 2006), and AvrPi-tz (Park et al., 2012), typically show high levels of sequence polymorphism in alleles that evade R protein detection. In contrast, Avr effectors that bind R-protein-guarded targets typically do not show high levels of sequence polymorphisms (they may be deleted or silenced in virulent strains of the pathogen). The high levels of sequence polymorphisms shown by Avr1b are thus more typical of proteins that interact directly with an R protein. Third, current models of R protein-guardee interactions propose that the guardee (here GmPUB1) holds the R protein (i.e., Rps1b) in an inactive state; when an effector such as Avr1b binds to the guardee disrupting its interaction with the R protein, the R protein is released into an active state and signals the activation of ETI. However, when we silenced the *GmPUB1* genes in the background of *Rps1*-b, the cotyledons became fully susceptible. Therefore, we propose a modified guard model, shown in Figure 7, in which Rps1-b is activated only when it recognizes avirulence alleles of Avr1b (from P6954 and P7063) in the context of GmPUB1. A variety of related models are also possible: for example, perhaps Rps1-b proteins can only recognize Avr1b proteins that have been ubiquitinated by GmPUB1; this would make GmPUB1 an “effector helper” protein as described by (Win et al., 2012a) rather than a guardee.

Despite extensive characterization of plant proteins and processes targeted by oomycete RxLR effectors, a systematic understanding of how the collection of RxLR effectors from an oomycete pathogen impact the immunity of its host remains poorly understood. The findings presented here suggest that there may be an extensive network of interactions involving multiple RxLR effectors and multiple plant U-Box E3 ligases (Supplementary Figure 3). Given the large numbers of E3 ligases encoded in plant genomes, and the complex sets of processes regulated by these proteins, the cumulative effect of numerous effector-E3 ligase interactions could profoundly re-program cell physiology. A major goal for the future research will be to finish mapping the network of effector E3 ligase interactions. This should encompass all families of E3 ligases on the host side and crinkler effectors as well as RxLR effectors on the pathogen side.

## Data Availability Statement

The original contributions (GSE182773) presented in the study are included in the article/Supplementary Material, further inquiries can be directed to the corresponding author/s.

## Author Contributions

SL characterized the interaction of Avr1b with GmPUB1 genes and wrote the first draft of the manuscript. RH designed and executed the Y2H experiments. HW designed and executed the cotyledon experiments and contributed to writing the manuscript. NP conducted the western blot analysis for determining the function of GmPUB1-1 protein. HB screened the yeast 2-hybrid library. CL conducted the double-barreled particle bombardment experiment to determine the function of GmPUB1. EP executed the cotyledon experiments. LZ designed and executed transcriptome experiments. HG constructed the yeast 2-hybrid library. BT planned the project, designed the experiments, analyzed the data, and wrote the manuscript. MB contributed to conception and design of the experiments and revised the manuscript. All authors contributed to the article and approved the submitted version.

## Funding

This work was supported in part by grants to MB from the Iowa Soybean Association and Agronomy Department, Iowa State University, in part by grants to BT from the National Science Foundation (IOS #0744875), from the Agriculture and Food Research Initiative of the USDA National Institute of Food and Agriculture (#2007-35319-18100 and #2010-65110-20764), and by support from Oregon State University.

## Conflict of Interest

The authors declare that the research was conducted in the absence of any commercial or financial relationships that could be construed as a potential conflict of interest.

## Publisher's Note

All claims expressed in this article are solely those of the authors and do not necessarily represent those of their affiliated organizations, or those of the publisher, the editors and the reviewers. Any product that may be evaluated in this article, or claim that may be made by its manufacturer, is not guaranteed or endorsed by the publisher.

## Supplementary Material

Supplementary Figure 1 RT-PCR analysis of the *GmPUB1* transcript levels following transformation of soybean cotyledons with *A. rhizogenes* harboring the GmPUB1 RNAi vector. Soybean Williams 82 cotyledonary tissues, different days following transformation with *A. rhizogenes* harboring the GmPUB1 RNAi vector, were harvested for preparation of total RNAs that were subjected to RT-PCR. Reduced GmPUB1 mRNA accumulation was observed on day 6 following transformation. Soybean *Actin* gene was used as the internal control to normalize the total RNAs used in RT-PCR analysis.

Supplementary Figure 2 Over-expression of GmPUB1-1 triggers cell death. Cell death was expressed as percentage survival of GUS-positive cells relative to a parallel empty vector control, using a double-barrel bombardment assay (Kale and Tyler, 2011). Lower percentages of surviving GUS-positive cells indicate increased cell death. The data means and standard errors were calculated from 7 to 8 bombardments per replicate; three replicates were conducted on Williams (*rps*) leaves, and one each on L77-1863 (*Rps1*-b) and Williams 82 (*Rps1*-k) leaves. Cell death due to GmPUB1-1 over-expression was significant (^**^*p* < 0.001) compared to empty vector for all three experiments, based on the Wilcoxon signed-ranks test (Kale and Tyler, 2011). There were no significant differences among soybean lines, based on the Wilcoxon rank sum test (*p* > 0.05) (Kale and Tyler, 2011).

Supplementary Figure 3 Yeast two-hybrid assays for interactions between RxLR effectors and PUB proteins. *RxLR* effector genes (lacking signal peptide coding regions) were cloned into pLAW10 and introduced into Y8930. *GmPUB* and *AtPUB* genes were cloned into pLAW11 and introduced into Y8800. The *RxLR*-containing strains were then mated with *PUB*-containing strains and plated on the indicated SD drop-out media. Strong interactions produce growth on –LTA while weak to medium interactions produce moderate or strong growth on –LTH plates, respectively.

Supplementary Table 1 Primers used for cloning soybean *GmPUB* genes and *P. sojae* effector genes into pLAW10 and pLAW11 plasmids (Spreadsheet).

Supplementary Table 2 The nucleotide sequences of primers used in pull down assay.

Supplementary Table 3 *GmPUB* gene expression levels during infection.
