# Supplementary figures and images for: Interaction of Phytophthora sojae Effector Avr1b With E3 Ubiquitin Ligase GmPUB1 Is Required for Recognition by Soybeans Carrying Phytophthora Resistance Rps1-b and Rps1-k Genes
Source: Front Plant Sci. 2021 Oct 6;12:725571. doi: 10.3389/fpls.2021.725571 (PMC8526854; doi:10.3389/fpls.2021.725571)

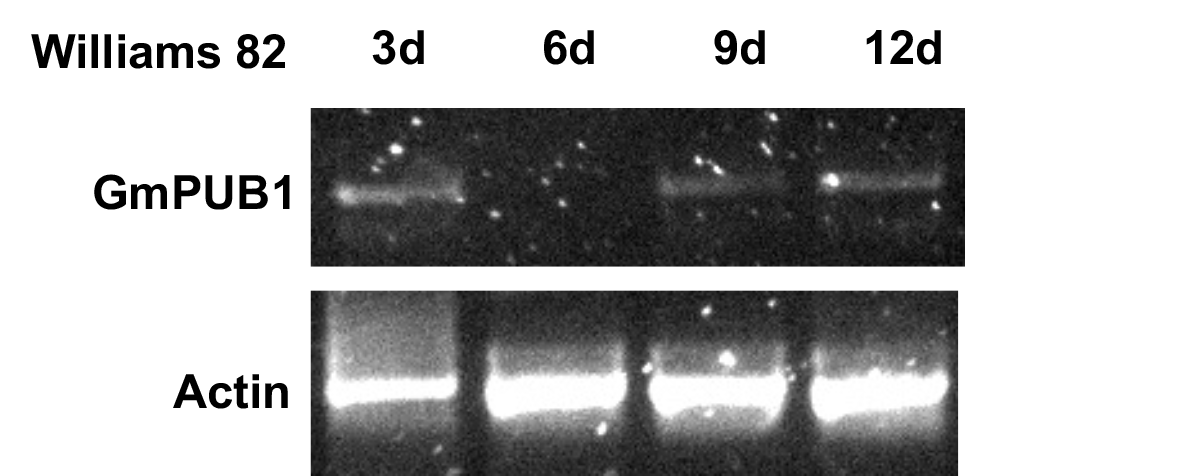

Supplement: Supplementary Figure 1 — RT-PCR analysis of the GmPUB1 transcript levels following transformation of soybean cotyledons with A. rhizogenes harboring the GmPUB1 RNAi vector. Soybean Williams 82 cotyledonary tissues, different days following transformation with A. rhizogenes harboring the GmPUB1 RNAi vector, were harvested for preparation of total RNAs that were subjected to RT-PCR. Reduced GmPUB1 mRNA accumulation was observed on day 6 following transformation. Soybean Actin gene was used as the internal control to normalize the total RNAs used in RT-PCR analysis. [file Image_1.TIF]

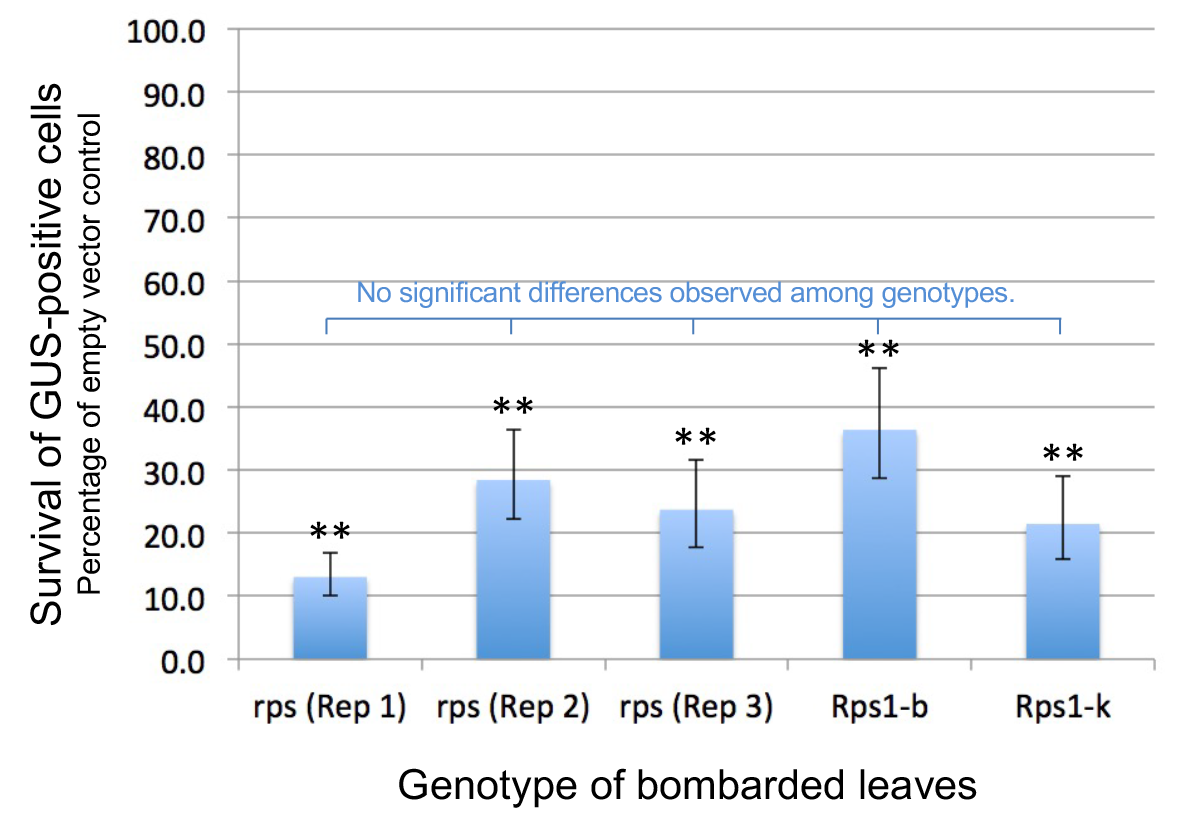

Supplement: Supplementary Figure 2 — Over-expression of GmPUB1-1 triggers cell death. Cell death was expressed as percentage survival of GUS-positive cells relative to a parallel empty vector control, using a double-barrel bombardment assay (Kale and Tyler, 2011). Lower percentages of surviving GUS-positive cells indicate increased cell death. The data means and standard errors were calculated from 7 to 8 bombardments per replicate; three replicates were conducted on Williams (rps) leaves, and one each on L77-1863 (Rps1-b) and Williams 82 (Rps1-k) leaves. Cell death due to GmPUB1-1 over-expression was significant (**p < 0.001) compared to empty vector for all three experiments, based on the Wilcoxon signed-ranks test (Kale and Tyler, 2011). There were no significant differences among soybean lines, based on the Wilcoxon rank sum test (p > 0.05) (Kale and Tyler, 2011). [file Image_2.TIFF]
