# Supplementary material for: Interaction of Phytophthora sojae Effector Avr1b With E3 Ubiquitin Ligase GmPUB1 Is Required for Recognition by Soybeans Carrying Phytophthora Resistance Rps1-b and Rps1-k Genes
Source: Front Plant Sci. 2021 Oct 6;12:725571. doi: 10.3389/fpls.2021.725571 (PMC8526854; doi:10.3389/fpls.2021.725571)
Supplement: Supplementary Table 2 — The nucleotide sequences of primers used in pull down assay. [file Table_2.docx]

**Table S2.** The nucleotide sequences of primers used in pull down assay.

| Target Sequence | Primer Name | | Sequences | |
| --- | --- | --- | --- | --- |
| HA-tagged Avr1b | T7 Kozak | CGAATTCTAATACGACTCACTATAGGGAACAGCCACCATGG | |  |
|  | Kozak_B42AD_HA | GGAACAGCCACCATGGCCTCCTACCCTTATGATG | |  |
|  | B42ADdT(30) | TTTTTTTTTTTTTTTTTTTTTTTTTTTTTTGGCAAGGTAGACAAGC | |  |
| His-tagged GmPUB1 | T7 Kozak | CGAATTCTAATACGACTCACTATAGGGAACAGCCACCATGG | |  |
|  | Kozak_pRSETA_His | GGAACAGCCACCATGGCCATGCGGGGTTCTCATC | |  |
|  | GmPUB1dT(29) | TTTTTTTTTTTTTTTTTTTTTTTTTTTTTCATGGATAGGAAGATAACAAAG | |  |
