# Supplementary material for: Interaction of Phytophthora sojae Effector Avr1b With E3 Ubiquitin Ligase GmPUB1 Is Required for Recognition by Soybeans Carrying Phytophthora Resistance Rps1-b and Rps1-k Genes
Source: Front Plant Sci. 2021 Oct 6;12:725571. doi: 10.3389/fpls.2021.725571 (PMC8526854; doi:10.3389/fpls.2021.725571)
Supplement: Supplementary Table 3 — GmPUB gene expression levels during infection. [file Table_3.docx]

**Table S3.** *GmPUB* gene expression levels during infection

| GmPUB family | Gene models | RPKM (infection) | RPKM (mock) | Ratio inf/mock | BH-adjusted  p value |
| --- | --- | --- | --- | --- | --- |
| GmPUB1-1  GmPUB1-2 | Glyma.13G312700.1 Glyma.12g189000.1 | 3.3 | 0.40 | 8.3 | 0.00046 |
| GmPUB2-1  GmPUB2-2 | Glyma.14G212200.1 Glyma.02G242900.1 | 40.1 | 0.63 | 64.0 | 0.00047 |
| GmPUB3-1  GmPUB3-2  GmPUB3-3 | Glyma.19G200200.1  Glyma.19G199300.1  Glyma.10G262600.1 Glyma.03G202600.1 | 17.3 | 0.52 | 33.0 | 0.0021 |
| GmPUB4-1  GmPUB4-2 | Glyma.12G188900.1 Glyma.13G312800.1 | 19.6 | 0.98 | 19.9 | 0.00049 |
| GmPUB5-1  GmPUB5-2 | Glyma.18G042100.1 Glyma.11G214500.1 | 39.5 | 2.87 | 13.8 | 0.00080 |
| GmPUB6-1  GmPUB6-2 | Glyma.09G172100.1 Glyma.07G106000.1 | 23.1 | 1.91 | 12.1 | 0.00046 |
| GmPUB7-1 | Glyma.02G195900.1 | 3.6 | 0.70 | 5.1 | 0.0034 |
| GmPUB8-1 | Glyma.02G196300.1 | 0.6 | 0.31 | 2.0 | 0.099 |
| GmPUB9-1  GmPUB9-2 | Glyma.U009600.1 Glyma.12G095000.1 | 6.6 | 0.12 | 56.3 | 0.010 |

Transcript levels *P. sojae*-infected and mock-inoculated Williams soybeans (12 h post inoculation) were determined by ABI Solid™ sequence tagging (Accession No. GSE182773), and quantitated as Reads Per Kilobase of transcript per Million mapped reads (RPKM). RPKM values were averaged for highly similar homeologs. Gene models correspond to Williams 82 assembly v2 annotation 1. *P* values were calculated by *t* tests of log-transformed RPKM values compared between infected and mock inoculated samples; a multiple test correction using the method of Benjamini and Hochberg (1995) was used.
